# Mechanoactivation of NOX2-generated ROS elicits persistent TRPM8 Ca^2+^ signals that are inhibited by oncogenic KRas

**DOI:** 10.1073/pnas.2009495117

**Published:** 2020-10-05

**Authors:** Stephen J. P. Pratt, Rachel M. Lee, Katarina T. Chang, Erick O. Hernández-Ochoa, David A. Annis, Eleanor C. Ory, Keyata N. Thompson, Patrick C. Bailey, Trevor J. Mathias, Julia A. Ju, Michele I. Vitolo, Martin F. Schneider, Joseph P. Stains, Christopher W. Ward, Stuart S. Martin

**Affiliations:** ^a^Program in Biochemistry and Molecular Biology, School of Medicine, University of Maryland, Baltimore, MD 21201;; ^b^Department of Pharmacology, School of Medicine, University of Maryland, Baltimore, MD 21201;; ^c^Department of Physiology, School of Medicine, University of Maryland, Baltimore, MD 21201;; ^d^Marlene and Stewart Greenebaum National Cancer Institute Comprehensive Cancer Center, School of Medicine, University of Maryland, Baltimore, MD 21201;; ^e^Department of Biochemistry and Molecular Biology, School of Medicine, University of Maryland, Baltimore, MD 21201;; ^f^Department of Orthopaedics, School of Medicine, University of Maryland, Baltimore, MD 21201;; ^g^School of Nursing, University of Maryland, Baltimore, MD 21201

**Keywords:** mechanotransduction, breast cancer, calcium, X-ROS; detyrosination

## Abstract

Changes in the mechanical microenvironment and mechanical signals are observed during tumor progression, malignant transformation, and metastasis. In this context, understanding the molecular details of mechanotransduction signaling may provide unique therapeutic targets. Here, we report that normal breast epithelial cells are mechanically sensitive, responding to transient mechanical stimuli through a two-part calcium signaling mechanism. We observed an immediate, robust rise in intracellular calcium (within seconds) followed by a persistent extracellular calcium influx (up to 30 min). This persistent calcium was sustained via microtubule-dependent mechanoactivation of NADPH oxidase 2 (NOX2)-generated reactive oxygen species (ROS), which acted on transient receptor potential cation channel subfamily M member 8 (TRPM8) channels to prolong calcium signaling. In contrast, the introduction of a constitutively active oncogenic KRas mutation inhibited the magnitude of initial calcium signaling and severely blunted persistent calcium influx. The identification that oncogenic KRas suppresses mechanically-induced calcium at the level of ROS provides a mechanism for how KRas could alter cell responses to tumor microenvironment mechanics and may reveal chemotherapeutic targets for cancer. Moreover, we find that expression changes in both NOX2 and TRPM8 mRNA predict poor clinical outcome in estrogen receptor (ER)-negative breast cancer patients, a population with limited available treatment options. The clinical and mechanistic data demonstrating disruption of this mechanically-activated calcium pathway in breast cancer patients and by KRas activation reveal signaling alterations that could influence cancer cell responses to the tumor mechanical microenvironment and impact patient survival.

## Significance

In breast cancer, the chronically stiffening mechanical microenvironment promotes tumor growth/invasion. However, the molecular details and integration of cancer mechanotransduction signaling pathways are not well understood. We find that nontumorigenic breast epithelial cells are mechanically sensitive and respond to acute mechanical stimuli with the generation of ROS-stimulated calcium signaling in a microtubule-dependent manner. These results establish that epithelial cells conserve a mechanotransduction pathway from muscle and bone (X-ROS). Using common breast cancer genetic mutations, we further report that oncogene activation reduces X-ROS pathway mechanoresponsiveness at the level of ROS sensitivity. This definition of how oncogene activation disrupts highly conserved mechanotransduction signaling mechanisms could improve the understanding of altered tumor cell responses to the mechanical microenvironment and reveal new therapeutic opportunities.

While cancer cells achieve uninhibited cell survival and increased capacity for metastasis through genetic instability, mounting evidence suggests that signaling from the cellular microenvironment can positively enhance these functions, including extracellular mechanical signals (reviewed in refs. 1–3). Prior to the onset of tumor formation, mammary stiffness and density are risk factors for malignant transformation (4, 5). During the progression of breast epithelial tumors, chronic alterations of mechanical signals and the mechanical microenvironment are observed; this includes increased stiffness (6–9), increased interstitial fluid pressure (10–12), increased solid stress (13, 14), and modified extracellular matrix (6, 15, 16). At the cellular level, the mechanical environment can be manipulated in vitro [i.e., two-dimensional (2D) or 3D stiff substrates] to either promote malignant transformation of normal mammary epithelial cells (17–20) or enhance malignant function of cancer cells [e.g., invasion (21), migration (22, 23), proliferation (24), and multinucleation (25)]. These long-term modifications to environmental mechanical properties highlight the role of mechanical signaling in cancer initiation and progression. However, activation of mechanotransducers and subsequent intracellular biochemical mechanotransduction pathways operate on much faster timescales, which are comparatively less studied in cancer biology and are often not integrated in studies on chronic remodeling of the tumor mechanical microenvironment. In nontumorigenic cells, some of the earliest signaling events that can occur in response to a mechanical stimulus are the unfolding of proteins (26–29), initiation of ionic currents (e.g., calcium) (30, 31), or generation of reactive oxygen species (ROS) signaling (32). Indeed, our prior work demonstrating a rapid calcium signaling response to scratch wounding in nontumorigenic breast epithelial and breast cancer cells supports a role for rapid mechanochemical signaling in breast cancer (33).

Rapid mechanical activation of ROS and calcium signaling (X-ROS) occurs in muscle (32, 34, 35) and bone (36); however, these early mechanically induced signaling pathways are generally unexplored in the epithelial cell types that give rise to carcinomas, which are responsible for more than 90% of human solid tumors (37). Defining the molecular mechanisms of rapid mechanochemical signaling in epithelial cells will help clarify how oncogene activation could alter interactions between cancer cells and the tumor mechanical microenvironment. Thus, we developed a method for tracking calcium signaling following acute and transient mechanical stimulation in mammary epithelial cells. This approach revealed alterations in calcium signals caused by oncogene expression. Specifically, we report here that human breast epithelial MCF10A cells initiate calcium signaling within seconds in response to a mechanical stimulus, but this calcium response remains persistent for at least 30 min. In contrast, overexpression of a constitutively active KRas oncogene inhibits the responsiveness to mechanical stimulation, by reducing the ability of cells to respond to the ROS component of the signaling pathway. We determined that the persistent intracellular calcium is sustained by the generation of ROS via reduced-form nicotinamide adenine dinucleotide phosphate (NADPH) oxidase 2 (NOX2), a process that is dependent on microtubules and consistent with the mechanotransduction signaling mechanisms defined in muscle and bone (32, 34–36). Moreover, data reveal that NOX2-generated ROS acts on transient receptor potential cation channel subfamily M member 8 (TRPM8) channels to prolong mechanically activated calcium. Further significance of this signaling pathway in cancer is highlighted by patient data which show that the combination of changes in NOX2/TRPM8 expression in estrogen receptor-negative (ER−) breast cancer patients is correlated with poor overall survival (hazard ratio, 2.4). We posit that these changes in the mechanical responsiveness of malignant cells by oncogenic KRas could distort the sensing of physical signals in the tumor microenvironment. Such alterations in rapid mechanotransduction by oncogenes could provide selective advantages to tumor cells during cancer progression.

## Materials and Methods

### Methods Summary.

A summary of materials and methods is outlined below. Detailed methods can be found in *SI Appendix*.

### Cell Culture, PTEN^−/−^ and KRas Mutations, and Reagents.

Human breast MCF10A epithelial cells were obtained from the American Type Culture Collection. The creation of cells and tumorigenic capacity are described in ref. 38, and metastatic capacity in ref. 39. The creation of the PTEN^−/−^ cells has been described previously (40, 41). Reagents used include 4 µM Fluo-4 AM (Life Technologies; F14201), hydrogen peroxide (H_2_O_2_) (Sigma-Aldrich; 21673), *N*-acetylcysteine (NAC) (USP Reference Standard 1009005), GP91ds-TAT (Anaspec; AS-63818) (42), colchicine (Sigma-Aldrich; C9754), and RQ-00203078 (Tocris; 5388).

### Mechanical Stimulation on Low Elastic Modulus Dishes and Dishes of Increasing Stiffness.

Mechanical stimulation on low elastic modulus dishes (mSLED) is possible when cells are plated on low elastic modulus substrates (0.2 kPa; Advanced Biomatrix; 5165); however, a subset of experiments used substrates of increasing stiffness (elastic moduli, 0.2, 0.5, 2, 8, 16, 32, and 64 kPa; Advanced Biomatrix; 5190) using identical methods otherwise. Glass capillaries were pulled and fire polished such that the end of the pipette tip was melted and sealed to a ∼160-µm rounded, bulbous end. As visualized in light microscopy, the glass pipette was used to compress the cell monolayer outside of the confocal scan area. Confocal time series imaging was immediately initiated and the pipette was maneuvered in the *x* direction until the pipette stimulus was applied across the entirety of the cell monolayer in view (similar to and adapted from a scratch assay, but does not create a scratch/gap area or induce cell damage). The pipette remained in its final place until completion of imaging.

### mSLED Optimization and Force Calculations.

mSLED stimulation involves both compression of the cell monolayer and simultaneous lateral movement of the pipette across the monolayer. In order to justify the degree of compression to achieve maximal calcium response, *Z*-stack images of mSLED stimulated cells were used to map the indentation of cell monolayer by pipette. The indentation depth of the cell monolayer was quantified using the difference between the average monolayer baseline (top) and bottom hundredth percentile of the indentation (bottom). Two depths of pipette compression (Normal vs. Shallow) were tested and compared with the respective calcium responses. In addition, forces applied to cells by pipette were calculated using the indentation depth (*d*), known elastic modulus of substrate (*E* = 0.2 kPa), and pipette radius (*R*) = 80 µm, using the Hertzian contact mechanics equation for a rigid sphere indenter (43): $F=\;\left(4/3\right)\left(E/\left(1-\upsilon^{2}\right)\right)R^{1/2}d^{3/2}$. The exact Poisson’s ratio of the highly elastic 0.2-kPa CytoSoft plates is unknown, so here we assume *ν* = 0.5 to calculate approximate forces.

### Kaplan–Meier Plotter.

The online Kaplan–Meier Plotter database (https://kmplot.com/analysis/) was used to test whether NOX2 or TRPM8 mRNA expression had effects on breast cancer patient clinical outcome (44, 45). The Affy id/Gene symbols used were NOX2 (203923_s_at), TRPM8 (243483_at), or both (“use multiple genes”).

### Statistics.

Statistical analyses were performed using MATLAB. For comparison between multiple conditions, a one-way ANOVA with a post hoc Tukey’s honest difference criterion was used and significance set at *P* < 0.05. For comparisons between pairs of conditions, a paired *t* test was used.

## Results

### mSLED: Mechanical Stimulation on Low Elastic Modulus Dishes.

**Fig. 1.**
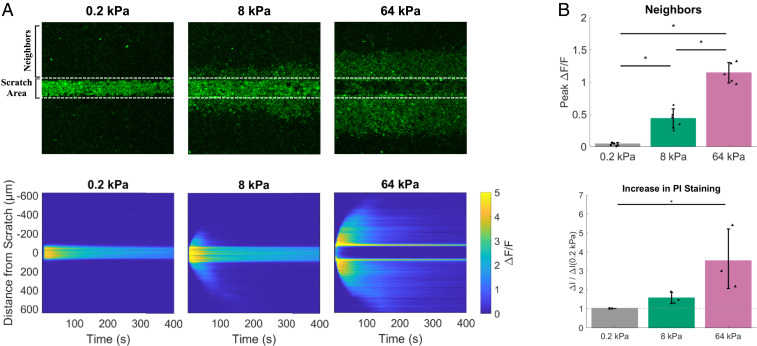
Stiffening of the extracellular microenvironment leads to cell damage-dependent intercellular signaling by mechanical stimulation. (*A*, *Top*) Scratch assays were applied to human breast epithelial cells (MCF10A) plated on soft (0.2 kPa), moderately stiff (8 kPa), and stiff (64 kPa) dishes. (*A*, *Bottom*) Kymographs show total scratch-induced calcium signaling (Δ*F*/*F*) signaling across the cell monolayer (0 ± 600 µm) as well as within the scratch area (0 ± 125 µm) during the experimental time course. Intercellular signaling to neighboring cells (neighbors) away from the scratch area (dotted lines) was only present in cells scratched on stiff (8 to 64 kPa) dishes and did not occur on soft dishes (0.2 kPa). (*B*, *Top*) Bar graph (Neighbors) shows quantification (peak Δ*F*/*F*) of signaling to neighbors from cells scratched on soft (0.2 kPa), moderately stiff (8 kPa), and stiff (64 kPa) dishes. Propidium iodide (PI) was used to measure cellular damage in scratched cells. (*B*, *Bottom*) Bar graph (increase in PI staining) shows quantification of changes in PI staining before vs. after scratch (Δ*I*/Δ*I*[0.2 kPa]). PI-positive cells were only present in cells scratched on stiff (8- to 64-kPa) dishes and were only present within the scratch area. Scratch on cells plated in soft environments (0.2 kPa) did not result in cellular damage as measured by PI. The collective data indicate that when cells plated on soft (0.2-kPa) substrates are stimulated in a manner that resembles a scratch wound assay, the stimulus does not create a scratch/gap area or induce cell damage-dependent signaling to neighboring cells, therefore resulting in a damage-independent calcium response. Data are presented as mean ± SD. The asterisk (*) indicates significance, which was set to *P* < 0.05 via one-way ANOVA with a post hoc Tukey’s honest difference criterion. For PI staining (Δ*I*/Δ*I*[0.2 kPa]), data represent *n* = 3 in total from three independent experiments for each group. For calcium signaling (Δ*F*/*F*), data represent *n* = 5 in total from five independent experiments for each group.

The tumor microenvironment demonstrates increased stiffening during in vivo tumor progression (6–9) (7), which can lead to increased cellular proliferation (24), migration (22), and invasion (21) during time courses ranging from 8 h to 5 d. However, the rapid mechanochemical signals are not well defined in epithelial tumor cells. Therefore, we developed an assay to test the earliest responsiveness of epithelial cells to mechanical stimuli. Our previously described real-time scratch assay (33) demonstrated the responsiveness of breast epithelial and breast cancer cells to acute mechanical scratch through the rapid initiation (within seconds) of cytoplasmic calcium signaling. However, scratch assays are routinely performed on rigid plastic or glass surfaces that greatly exceed the stiffness of the tumor microenvironment, are inherently damaging to cells, and can introduce confounding elements resulting from the nonspecific dumping of cellular contents. We instead performed mechanical scratch on surfaces with a range of stiffnesses that more closely match the mechanical microenvironment of the normal mammary gland, as well as stiffnesses more characteristic of malignant tissue (9). By applying the scratch assay on dishes of different stiffness, we were able to isolate nondamaging from damaging mechanical stimuli (Fig. 1 and *SI Appendix*, Figs. S1–S3). Costaining with propidium iodide (PI) showed that when membrane integrity was compromised (PI-positive staining), calcium signaling occurred in neighboring cells away from the scratch area, which was an effect primarily present in the stiff dishes but not in the soft dishes (Fig. 1 *A* and *B* and *SI Appendix*, Figs. S2 *A* and *B* and S3 *A*–*C*). The high correlation (0.86) between substrate stiffness and PI staining (*SI Appendix*, Fig. S3 *C*, *Bottom Right*), as well as a high correlation (0.88) between PI staining and calcium signaling in neighboring cells (*SI Appendix*, Fig. S3 *C*, *Top Right*), indicates that loss of membrane integrity occurs in the mechanically scratched area on stiffer surfaces and generates a damage-dependent signal necessary for activation of calcium in neighboring cells.

**Fig. 2.**
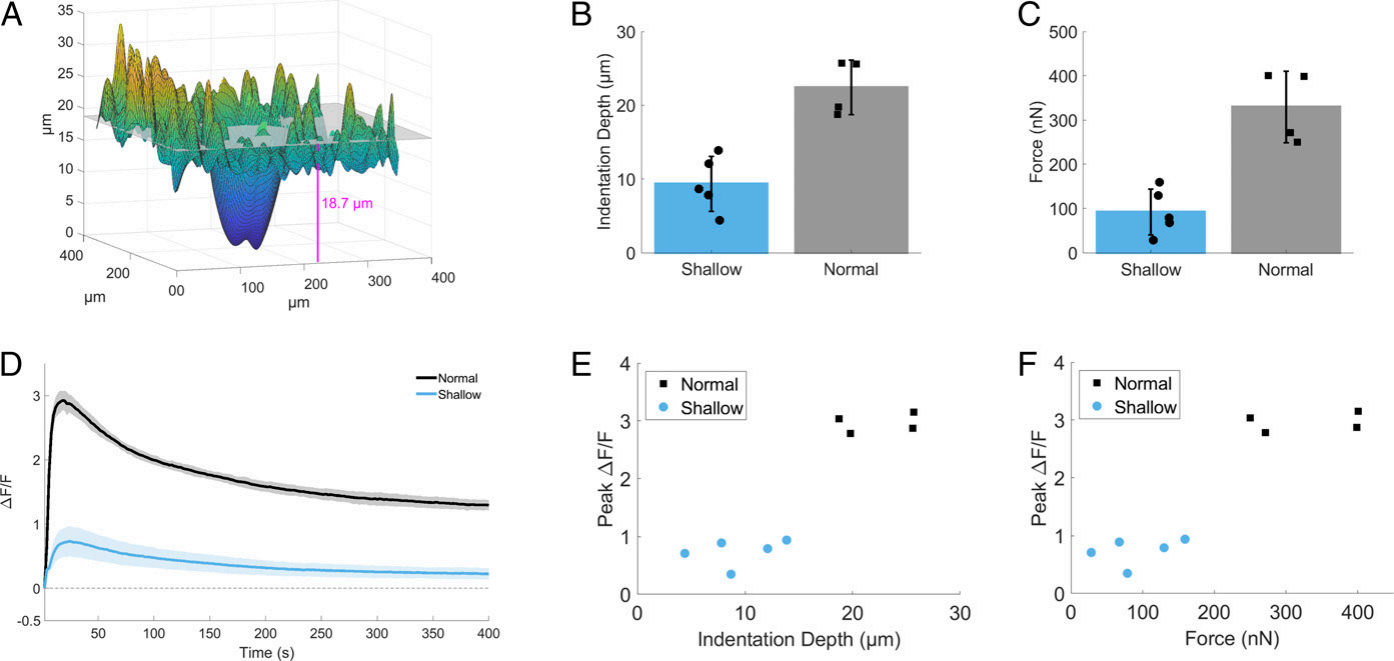
mSLED optimization and force calculations. (*A*) *Z*-stack images of mSLED-stimulated cells were used to map the indentation of the cell monolayer by pipette. The indentation depth of the cell monolayer was quantified using the difference between the average monolayer baseline (*Top*) and bottom hundredth percentile of the indentation (*Bottom*). In addition, forces applied by pipette onto cells were calculated using the indentation depth (*d*), known elastic modulus of substrate (*E* = 0.2 kPa), and pipette radius (*R*) = 80 µm, using the Hertzian contact mechanics equation for a rigid sphere indenter $F=\;\left(4/3\right)\left(E/\left(1-\upsilon^{2}\right)\right)R^{1/2}d^{3/2}$. (*B* and *C*) Two depths of pipette compression were tested (Normal vs. Shallow), which show average differences in indentation depth (Normal, 22.5 ± 3.7 µm; Shallow, 9.4 ± 3.7 µm) and average force applied to cells (Normal, 330.1 ± 80.9 nN; Shallow, 92.7 ± 51.8 nN). (*D*) Calcium signaling in response to alternate modes of mSLED stimulation resulted in different relative changes in peak fluorescence (peak Δ*F*/*F*) (Normal, 2.9 ± 0.16, vs. Shallow, 0.74 ± 0.23) and persistent fluorescence (Δ*F*/*F* at 6 min) (Normal, 1.3 ± 0.09, vs. Shallow, 0.24 ± 0.09). (*E* and *F*) A difference in average indentation depth of ∼13.1 µm, or average force of ∼237.3 nN, resulted in a threefold increase in intracellular calcium signaling.

When cells are stimulated by the pipette on dishes with a low elastic modulus, which better matches the mechanical environment of the mammary gland (0.2 and 0.5 kPa) (9), we find a lack of a gap/scratch in the cell monolayer, cell damage, or cell damage-dependent intercellular signaling to neighboring cells (Fig. 1 and *SI Appendix*, Figs. S2 and S3). Due to the elasticity of the substrate, mechanical stimulation involves slight compression of the cell monolayer (Fig. 2*A*) as well as simultaneous lateral movement of the pipette for maximal calcium response (therefore resembling a “scratch wound assay,” but does not induce a cell damaging wound). Two depths of pipette compression were tested (Fig. 2*B*; Normal, 22.5 ± 3.7 µm, vs. Shallow, 9.4 ± 3.7 µm) and were compared with the respective forces applied to cells (Fig. 2*C*; Normal, 330.1 ± 80.9 nN, vs. Shallow, 92.7 ± 51.8 nN) and calcium responses (Fig. 2*D*). In order to initiate robust intracellular calcium signaling (Peak Δ*F*/*F*: Normal, 2.9 ± 0.16, vs. Shallow, 0.74 ± 0.23; and Δ*F*/*F* at 6 min: Normal, 1.3 ± 0.09, vs. Shallow, 0.24 ± 0.09), a difference in average indentation depth of ∼13.1 µm, or average force of ∼237.3 nN, was necessary (Fig. 2 *E* and *F*). While the specific in vivo dynamic mechanical stresses of the breast have not been clarified to date, the average force applied of 330.1 ± 80.9 nN agrees well with physiologic ranges at the level of the cell. For example, cell–cell contacts between two epithelial cells can generate forces up to 400 nN (46), the contractile actomyosin cortex during mitosis in HeLa cells can generate a surface tension between 0.2 and 1.6 mN/m (47), and single cardiomyocyte contractile force can exceed 3 μN (48).

**Fig. 3.**
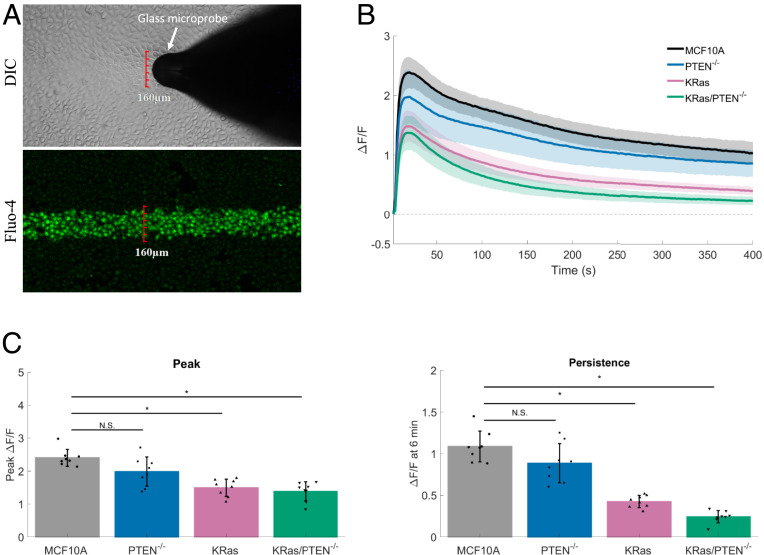
Constitutively active KRas but not PTEN loss reduces mechanoresponsiveness in MCF10A breast epithelial cells. (*A*) Mechanical stimulation of cells plated on 0.2-kPa substrates, termed mSLED (mechanical stimulation on low elastic modulus dishes), shows that only cells within the 160-µm-wide stimulus area (DIC) respond with rises in intracellular calcium (Fluo-4). (*B*) Traces show mechanically stimulated calcium signaling (Δ*F*/*F*) plotted over time for human breast epithelial cells (MCF10A), MCF10A PTEN null mutant cells (PTEN^−/−^), MCF10A constitutively active KRas (G12V) mutant cells (KRas), or MCF10A combination mutant cells (KRas/PTEN^−/−^). (*C*) Bar graphs show quantification of peak Δ*F*/*F* and Δ*F*/*F* at 6 min (persistence). The KRas and KRas/PTEN^−/−^ mutation, but not PTEN loss alone, resulted in significant reductions in peak Δ*F*/*F* and Δ*F*/*F* at 6 min. Data are presented as mean ± SD. The asterisk (*) indicates significance, which was set at *P* < 0.05 via one-way ANOVA with a post hoc Tukey’s honest difference criterion. N.S. indicates no significant difference. Data represent *n* = 6 in total from six independent experiments for each group.

Mechanical stimulation of cells on 0.2-kPa substrates results in the activation of calcium only in the cells that have been stimulated with the glass microprobe (∼160-µm-wide fluorescent stripe) (Fig. 3*A*) (Movie S1). This mechanical stimulus initiates rapid (within seconds) changes in Fluo-4 fluorescence (peak Δ*F*/*F* = 2.1 ± 0.2), which persists for long periods of time (up to 30 min). The majority of the calcium signaling occurs within this 30-min time period (*SI Appendix*, Fig. S4*A*). Even at these later time points, there was also no neighboring cell calcium signaling or increase in PI-positive staining (*SI Appendix*, Fig. S4*B*). The pipette stimulation of cells plated on dishes of low elastic modulus in the absence of cellular damage, now termed mSLED, is a method of mechanically stimulating cells, and when coupled with calcium imaging, can serve to measure mechanoresponsiveness in breast epithelial cells. We additionally compared our method to other common approaches for application of mechanical stress. We surmised that fluid flow would be an appropriate additional method to test the rapid mechanical responsiveness of breast epithelial cells to physiologic mechanical stress. Indeed, MCF10A cells similarly respond to fluid flow with rapid intracellular calcium signaling (*SI Appendix*, Fig. S5). However, fluid flow plate reader assays limit the ability to simultaneously perform high-resolution confocal imaging.

### Constitutively Active KRas but Not PTEN Loss Reduces Mechanoresponsiveness in MCF10A Breast Epithelial Cells.

To examine how oncogene expression affects rapid mechanotransduction in cancer cells, we utilized the mSLED approach in nontumorigenic MCF10A cells (control) or a series of genetically-transformed MCF10A cells. We have previously engineered normal breast epithelial MC10A cells with homozygous deletion of the PI3K-counteracting PTEN phosphatase, overexpression of activated KRas(G12V), or in combination (KRas/PTEN^−/−^) (38). KRas mutations and PTEN loss confer to nontumorigenic MCF10A cells the ability to persist in vivo, while KRas/PTEN^−/−^ cells form robust tumors (38). However, about 50% of the KRas group are able to form delayed tumors (8 wk), while the PTEN^−/−^ group has the inability for tumor outgrowth (measured up to 2 y). Moreover, in vivo models for metastasis show that a gain of KRas mutation increases survival of MCF10A cells in the lung compared to PTEN loss, but a combined KRas/PTEN^−/−^ mutation yields rapid metastatic outgrowth that significantly precedes each individual mutation (39). The collective data suggest that PTEN mutations confer apoptotic resistance, while KRas mutations confer a growth advantage, and the combination yields the capacity for unchecked tumor formation. Two major strengths of these four cell lines are that they model tumorigenicity and metastasis in a stepwise fashion using commonly deregulated signaling pathways in breast cancer, and they eliminate confounding effects of the numerous additional mutations in common breast cancer cell lines used for research. Such a stepwise model yields the ability to investigate mechanisms linking specific oncogenic mutations to cancer phenotypes such as changes in mechanoresponsiveness. Therefore, human breast epithelial cells (MCF10A), MCF10A PTEN-null mutant cells (PTEN^−/−^), MCF10A constitutively-active KRas mutant cells (KRas), and MCF10A combination mutant cells (KRas/PTEN^−/−^) were plated and mechanically stimulated via mSLED (Fig. 3 *B* and *C*). Both KRas and KRas/PTEN^−/−^ cells showed significant reductions in the magnitude of initial calcium influx compared with MCF10A, but KRas and KRas/PTEN^−/−^ cells also severely blunted the persistent calcium signaling (Fig. 3*C*). No significant differences in initial or persistent calcium signaling were observed between MCF10A and PTEN^−/−^ cells. These data indicate that constitutively active KRas, but not PTEN loss, reduces mechanical responsiveness of normal breast epithelial cells.

### Microtubule-Dependent Mechanically Activated NOX2-Derived ROS Sustains Calcium Signaling.

**Fig. 4.**
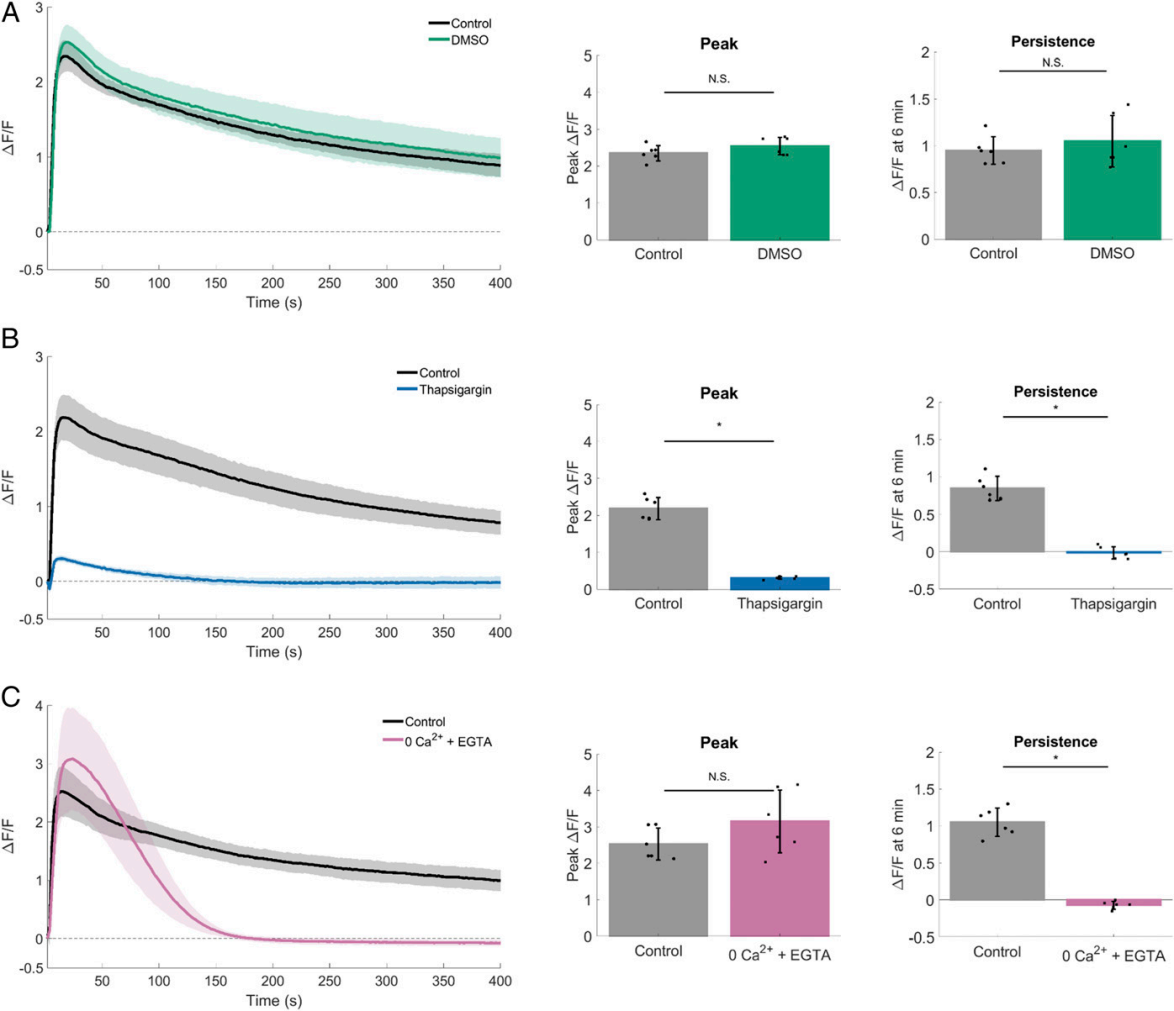
Intracellular and extracellular calcium stores are necessary for mechanically stimulated calcium signaling. For each dataset, traces show mechanically stimulated calcium signaling (Δ*F*/*F*) plotted over time for control and treated cells. Bar graphs show quantification of peak Δ*F*/*F* and Δ*F*/*F* at 6 min (persistence). (*A*) Quantification (peak Δ*F*/*F* and Δ*F*/*F* at 6 min) revealed rapid mechanically stimulated increases in intracellular calcium that persist for longer periods of time. DMSO had no effect on calcium signaling. (*B*) Compared with control, peak Δ*F*/*F* and Δ*F*/*F* at 6 min was blocked in groups depleted of intracellular calcium (2 µM thapsigargin). (*C*) Depletion of external calcium (0Ca^2+^ + EGTA) did not significantly change peak Δ*F*/*F* but blocked Δ*F*/*F* at 6 min. Data are presented as mean ± SD. The asterisk (*) indicates significance from control, *P* < 0.05 via paired *t* test. N.S. indicates no significant difference. Data represent *n* = 6 in total from six independent experiments for each group. Control groups were collected separately for each dataset.

Given that KRas oncogene activation was solely sufficient to reduce the mechano-dependent persistent calcium response (Fig. 3 *B* and *C*), we sought to define the molecular mechanisms that underlie this effect. To determine the source of calcium responsible for this persistence (Fig. 4), we first depleted intracellular calcium from the responsive MCF10A parental cells using thapsigargin, which binds to the sarco-endoplasmic calcium ATPase (SERCA) preventing calcium reuptake to the endoplasmic reticulum (ER). Vehicle control applied up to 5% DMSO had no significant effect on mSLED-stimulated calcium signaling (Fig. 4*A*). In contrast, thapsigargin (0.2% DMSO) effectively blocked both initial and persistent calcium signaling (Fig. 4*B*). Therefore, the initiation of the mechano-dependent calcium signaling requires intracellular stores from the ER.

We next depleted extracellular calcium using calcium-free imaging media supplemented with EGTA (0Ca^2+^ + EGTA). In contrast to treatment with thapsigargin, depletion of extracellular calcium only inhibited persistent calcium and was not required for initial peak calcium (Fig. 4*C*). Thus, the persistent calcium signal, which can be reduced with KRas oncogene activation, is dependent on calcium flux across the plasma membrane. These combined data support a basic mechanism where the initial mechanically activated peak signal requires intracellular calcium, but extracellular calcium is necessary for persistent calcium signaling.

**Fig. 5.**
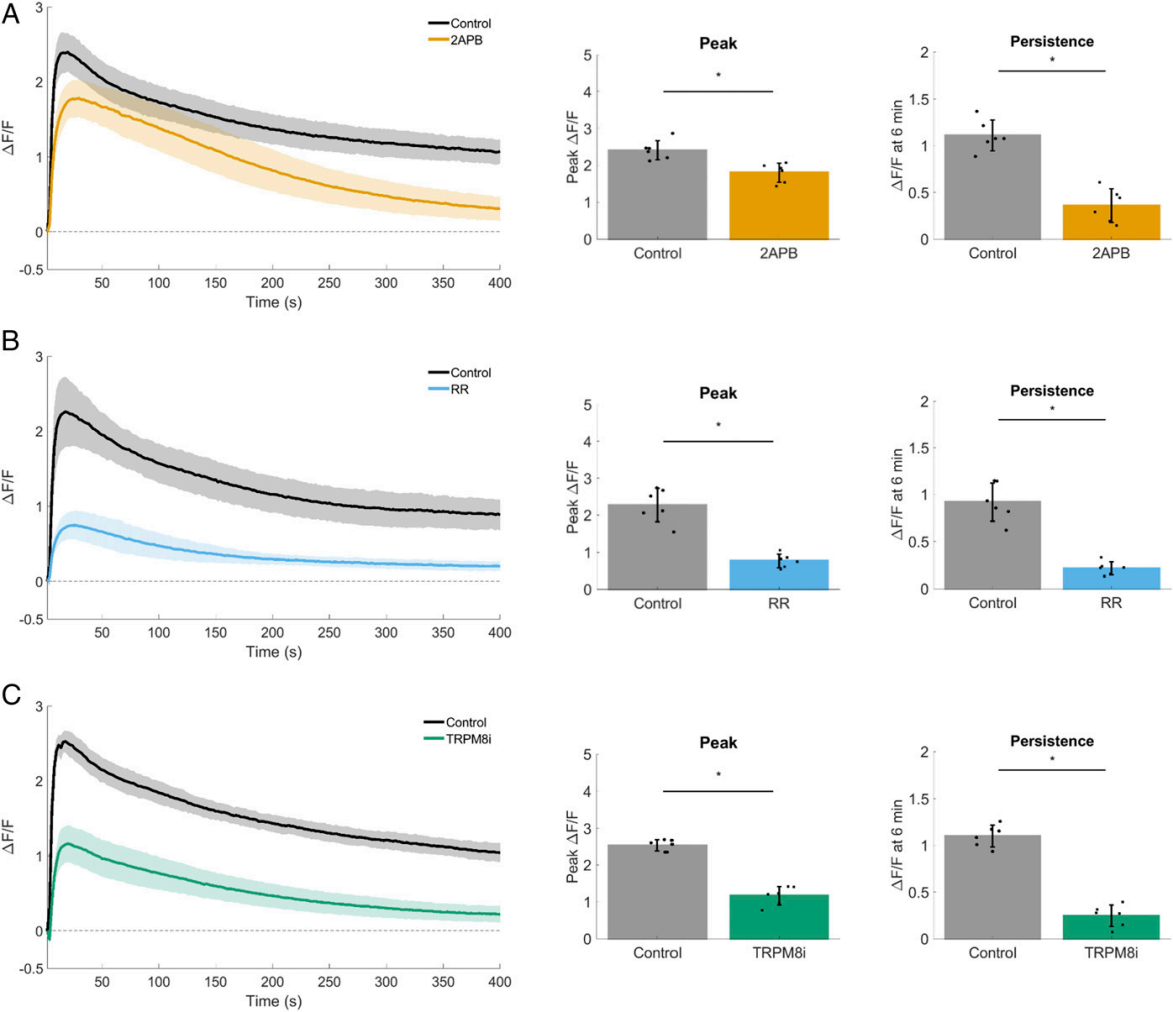
TRPM8 is necessary for mechanically stimulated calcium signaling. For each dataset, traces show mechanically stimulated calcium signaling (Δ*F*/*F*) plotted over time for control and treated cells. Bar graphs show quantification of peak Δ*F*/*F* and Δ*F*/*F* at 6 min (persistence). (*A*) 2-Aminoethoxydiphenylborane (2-APB) inhibits store operated calcium release (SOCE) and inositol 1,4,5-trisphosphate (IP3) receptors, but can also act as a nonspecific TRP channel blocker. Treatment with 2-APB (200 µM) resulted in a significant reduction of peak Δ*F*/*F*, with the largest inhibition seen in Δ*F*/*F* at 6 min. (*B*) Ruthenium Red (RR) (30 µM) is also a nonselective TRP channel blocker. RR inhibited both peak Δ*F*/*F* and Δ*F*/*F* at 6 min compared with control. (*C*) RQ-00203078 is a potent and selective transient receptor potential cation channel subfamily M member 8 (TRPM8) antagonist (TRPM8i). RQ-00203078 (25 µM) inhibited both peak Δ*F*/*F* and Δ*F*/*F* at 6 min compared with control. Data are presented as mean ± SD. The asterisk (*) indicates significance from control, *P* < 0.05 via paired *t* test. Data represent *n* = 6 in total from six independent experiments for each group. Control groups were collected separately for each dataset.

Mechanically-activated calcium influx across the plasma membrane typically arise via ion channels from the Piezo or TRP protein families (reviewed in ref. 3). To test whether TRP channels in general are necessary for mSLED-stimulated calcium signaling in MCF10A cells, nonselective TRP inhibitors 2-aminoethoxydiphenylborane (2-APB) and ruthenium red (RR) were used (Fig. 5 *A* and *B*). Both 2-APB and RR blocked calcium at 6 min, supporting a role for TRP channels in persistent calcium from MCF10A cells. TRPM8 is expressed in human breast tissue and its expression is increased in breast tumors (49, 50); therefore, we aimed to test the specific role of TRPM8. Furthermore, a potent and selective (51–53) TRPM8 antagonist (RQ-00203078) is readily available. MCF10A cells were incubated with RQ-00203078 and subjected to mSLED. Compared to control, RQ-00203078 (TRPM8i) significantly blocked initial and persistent calcium (Fig. 5*C*). These combined data (2-APB, RR, RQ-00203078) show that the plasma membrane channel TRPM8 is required for mechanically-activated calcium influx.

**Fig. 6.**
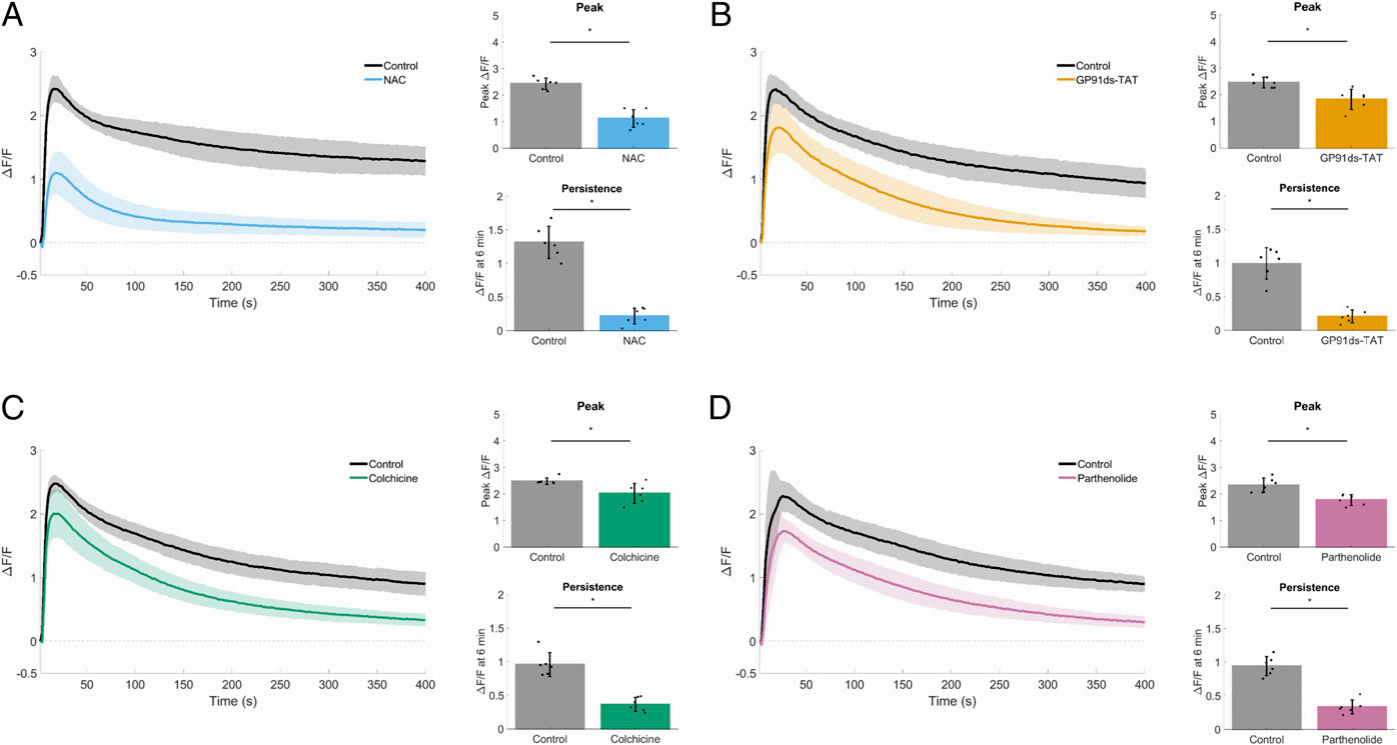
Microtubule-dependent mechanically activated NOX2-derived ROS sustains calcium signaling. For each dataset, traces show mechanically stimulated calcium signaling (Δ*F*/*F*) plotted over time for control and treated cells. Bar graphs show quantification of peak Δ*F*/*F* and Δ*F*/*F* at 6 min (persistence). (*A*) The reactive oxygen species (ROS) scavenger *N*-acetylcysteine (NAC) (40 mM) inhibited both peak Δ*F*/*F* and Δ*F*/*F* at 6 min compared with control. (*B*) The specific peptide inhibitor (GP91ds-TAT; 1.5 µM) of NADPH oxidase 2 (NOX2) resulted in a significant reduction of peak Δ*F*/*F*, with the largest inhibition seen in Δ*F*/*F* at 6 min. (*C*) The microtubule depolymerizer colchicine (200 µM) resulted in a significant reduction of peak Δ*F*/*F*, with the largest inhibition seen in Δ*F*/*F* at 6 min. (*D*) Inhibition of the stabilizing microtubule posttranslational modification detyrosination using parthenolide (200 µM) resulted in a significant reduction of peak Δ*F*/*F*, with the largest inhibition seen in Δ*F*/*F* at 6 min. The collective data identify the key components of the microtubule-dependent NOX2-ROS mechanotransduction pathway (i.e., X-ROS) and are necessary for sustaining long-term (persistent) mechanically activated calcium. Data are presented as mean ± SD. The asterisk (*) indicates significance from control, *P* < 0.05 via paired *t* test. Data represent *n* = 6 in total from six independent experiments for each group. Control groups were collected separately for each dataset.

To examine mechanisms upstream of TRPM8 activation, we were informed by studies in muscle (32, 34, 35) and bone (36) establishing a microtubule-dependent mechanotransduction mechanism through NOX2-generated ROS as an activator of TRP calcium channels. A brief incubation with the ROS scavenger *N*-acetylcysteine (NAC) blocked mechanically-activated calcium signaling (Fig. 6*A*), implicating ROS as an upstream activator. Cells were then treated with a specific peptide inhibitor, GP91ds-TAT, preventing the association of NOX2 (gp91^phox^) and the activating subunit p47^phox^ (42). Inhibition of NOX2 resulted in a significant reduction in persistent calcium (Fig. 6*B*), therefore indicating NOX2 as the source of ROS.

**Fig. 7.**
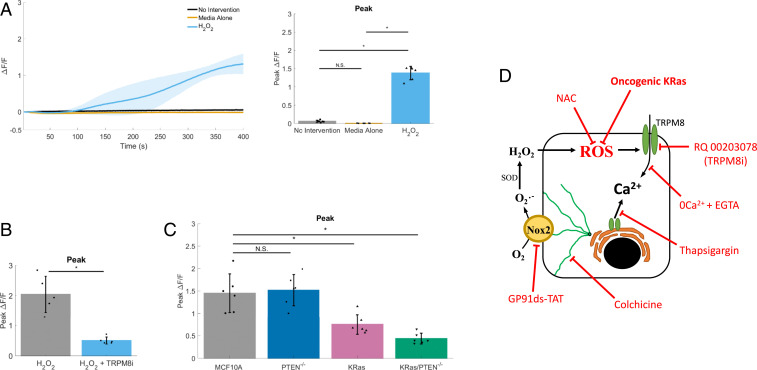
ROS activates calcium signaling through TRPM8, but ROS sensitivity is inhibited in KRas mutant cells. (*A*) Traces show reactive oxygen species (ROS)-stimulated calcium signaling (Δ*F*/*F*) plotted over time. Bar graph shows quantification of peak Δ*F*/*F*. During imaging, 1 mL of 20 mM hydrogen peroxide (positive control for ROS, “H_2_O_2_”) was added to cells containing 1 mL of media (final H_2_O_2_ concentration, 10 mM) and compared to either addition 1 mL of media (“Media Alone”) or no intervention (“No Intervention”). Addition of H_2_O_2_ resulted in a gradual increase in intracellular calcium, compared to negative controls. Data are presented as mean ± SD. The asterisk (*) indicates significance from control, *P* < 0.05 via paired *t* test. N.S. indicates no significant difference. Data represent *n* = 6 in total from six independent experiments for each group. (*B*) Bar graph shows quantification of peak Δ*F*/*F*. During imaging, 1 mL of 20 mM hydrogen peroxide (positive control for ROS) was added to cells containing 1 mL of control media (H_2_O_2_) or 25 µM RQ-00203078, a potent and selective TRPM8 antagonist (H_2_O_2_ + TRPM8i) (final H_2_O_2_ concentration, 10 mM). Addition of H_2_O_2_ in RQ-00203078–treated cells showed a reduced peak (Δ*F*/*F*) ROS-induced calcium compared with controls. Data are presented as mean ± SD. The asterisk (*) indicates significance from control, *P* < 0.05 via paired *t* test. Data represent *n* = 6 in total from six independent experiments for each group. (*C*) Bar graph shows quantification of peak Δ*F*/*F*, for ROS-stimulated intracellular calcium (Δ*F*/*F*) over time in human breast epithelial cells (MCF10A), MCF10A PTEN null mutant cells (PTEN^−/−^), MCF10A constitutively active KRas(G12V) mutant cells (KRas), or MCF10A combination mutant cells (KRas/PTEN^−/−^). Compared with control MCF10A parental cells, addition of H_2_O_2_ resulted in a significantly reduced ROS-stimulated intracellular calcium in KRas or KRas/PTEN^−/−^ mutants. Data are presented as mean ± SD. The asterisk (*) indicates significance, which was set at *P* < 0.05 via one-way ANOVA with a post hoc Tukey’s honest difference criterion. N.S. indicates no significant difference. Data represent *n* = 6 in total from six independent experiments for each group. (*D*) Graphic summarizes data showing mechanisms of mechanically induced X-ROS signaling in normal breast MCF10A epithelial cells. Cell damage-independent mechanical stimulation on low elastic modulus dishes (mSLED) mechanically stimulates microtubules to activate NADPH oxidase 2 (NOX2), which generates reactive oxygen species (ROS). ROS in turn acts on calcium channels such as TRPM8 to sustain long-term (persistent) mechanically activated calcium. However, overexpression of a constitutively active KRas oncogene inhibits the responsiveness to mechanical stimulation by reducing the ability of cells to respond to the ROS component of the signaling pathway. Pharmacological inhibitors or oncogenic activation used to elucidate mechanism are shown in red. The arrows indicate positive action or induction. The bar-headed lines indicate inhibition. 0Ca^2+^ + EGTA, depletes extracellular calcium and effectively inhibits calcium flux across the plasma cell membrane; colchicine, binds α-tubulin to effectively depolymerize microtubules; GP91ds-TAT, inhibitory peptide against NOX2; H_2_O_2_, hydrogen peroxide, positive control for ROS; NAC, *N*-acetylcysteine, an antioxidant or ROS negative control; oncogenic KRas, overexpression of activated KRas (G12V) in normal MCF10A cells; RQ-00203078/TRPM8i, a potent and selective transient receptor potential cation channel subfamily M member 8 (TRPM8) antagonist; thapsigargin, binds sarco/endoplasmic reticulum Ca^2+^-ATPase (SERCA) and effectively depletes intracellular calcium and inhibits calcium flux from the endoplasmic reticulum.

The mechanoactivation of NOX2 requires microtubules that are posttranslationally modified by detyrosination (34–36). Consistent with this microtubule-dependent mechanotransduction, we show that treatment with colchicine to depolymerize microtubules or parthenolide to reduce detyrosination inhibited persistent calcium in a similar fashion to blocking NOX2 (Fig. 6 *C* and *D*). Taken together, these results show that the microtubule-dependent mechanotransduction through NOX2 is necessary for mSLED-induced persistent calcium, but whether the ROS signaling is solely sufficient to activate TRPM8 was unclear. To investigate, hydrogen peroxide (H_2_O_2_) was directly applied to Fluo-4–loaded MCF10A cells to test for calcium activation by ROS. H_2_O_2_ increased peak Δ*F*/*F* compared with controls (addition of media alone or no intervention) (Fig. 7*A*). To specifically test whether ROS converges on TRPM8 to initiate calcium signaling, H_2_O_2_ was used to activate calcium in the presence of the TRPM8 inhibitor compared with control (Fig. 7*B*). Indeed, RQ-00203078 showed a large and significant inhibition of H_2_O_2_-induced calcium signaling.

### KRas Mutants Inhibit Mechanoresponsiveness via Reduced ROS Sensing.

Since KRas mutation can inhibit mechanically-stimulated calcium (Fig. 3), we leveraged the mechanisms that we defined in MCF10A cells to identify how this pathway is inhibited by KRas. Mechanical stimulation of MCF10A mammary epithelial cells results in activation of microtubule-dependent NOX2 to generate ROS, which in turn acts on TRPM8 channels to prolong calcium signaling. One mechanism by which KRas activation disrupts this pathway could be altered calcium signaling activation by ROS, since tumor cells commonly have disrupted redox states. To measure calcium activation by H_2_O_2_ in normal cells vs. cells with oncogenic mutations, we applied H_2_O_2_ to Fluo-4–loaded MCF10A, PTEN^−/−^, KRas, and KRas/PTEN^−/−^ cells (Fig. 7*C*). Compared to MCF10A or PTEN^−/−^ cells, KRas and KRas/PTEN^−/−^ cells showed a significant reduction in H_2_O_2_-dependent Δ*F*/*F* (Fig. 7*C*). Similar to the observed reductions in mechanoresponsiveness by KRas activation, we see that PTEN^−/−^ mutations have no effect on H_2_O_2_-stimulated calcium, but KRas mutations inhibit these signals.

Our collective data demonstrate that when mechanical stimulation of mammary epithelial cells can be isolated from cell damage-induced signals, this mechanical stimulation results in activation of microtubule-dependent NOX2 to generate ROS, which in turn acts on TRPM8 channels to prolong calcium signaling (Fig. 7*D*). However, KRas and KRas/PTEN^−/−^ cells greatly reduce this persistent calcium in response to mechanical cues and are insensitive to H_2_O_2_-induced activation of calcium. Furthermore, there are no discernible differences in TRPM8 expression between the MCF10A variant cell lines (*SI Appendix*, Fig. S6). The data support a reduction in mechanically-stimulated persistent calcium by KRas and further indicate that the KRas inhibition of mechanoresponsiveness specifically occurs at the level of ROS upstream of TRPM8. This is further supported by the notion that cancer cells have the increased capacity to detoxify ROS (54). The consequences of this loss of mechanosensitivity and insensitivity to ROS-activated persistent calcium may contribute to changes in tumor cell behavior that could eventually be exploited for therapeutic gain.

### NOX2 and TRPM8 mRNA Expression Predicts Patient Overall Survival.

**Fig. 8.**
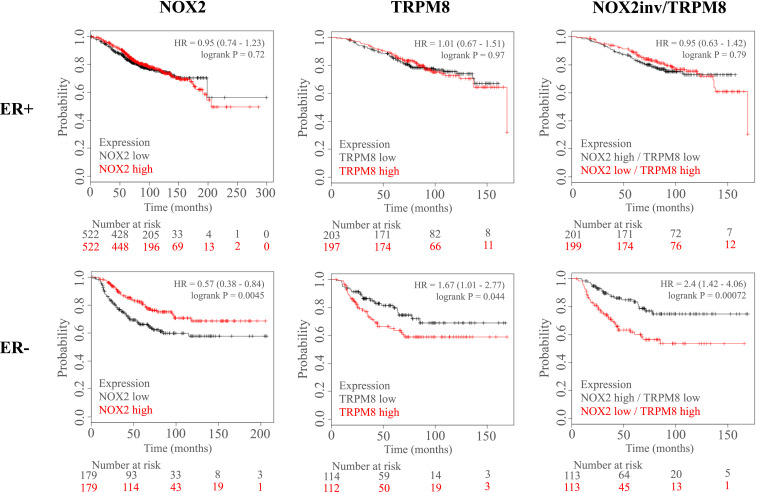
NOX2 and TRPM8 mRNA expression effect estrogen receptor negative patient median overall survival. The Kaplan–Meier Plotter database (KMplotter) was used to test whether components of the mechanically sensitive pathway identified in normal breast MCF10A epithelial cells had an effect on breast cancer patient clinical outcome. Overall survival probability was plotted against time (months) for patients with high vs. low mRNA expression of NOX2 (*Left*) or TRPM8 (*Middle*) and were also separated by estrogen receptor-positive (ER+) (*Top*) vs. estrogen receptor-negative (ER−) (*Bottom*) status. NOX2 and TRPM8 expression showed no difference in overall survival of ER+ breast cancer patients. Conversely, in ER− patients, NOX2 and TRPM8 showed an inverse outcome on overall survival whereby patients with high expression of NOX2 showed greater survival (hazard ratio, 0.57) and patients with high expression of TRPM8 showed poor survival (hazard ratio, 1.67). Therefore, the combination of NOX2 and TRPM8 mRNA expression was tested for effects on overall survival, but data were separated using inverted NOX2 expression data and unchanged TRPM8 data. The data show that the combination of low NOX2 mRNA and high TRPM8 mRNA was present in ER− patients with poor clinical outcome (hazard ratio, 2.4) vs. patients with NOX2 high/TRPM8 low. The Affy id/Gene symbol used for NOX2 and TRPM8 are (203923_s_at) and (243483_at), respectively. Patient data were split by the median expression level for each gene. Number at risk refers to the number of patients included in each time point.

The Kaplan–Meier Plotter database (KMplotter) was used to test whether key components of this mechanically sensitive calcium pathway have effects on breast cancer patient clinical outcome. Overall survival probability was plotted for patients with high vs. low mRNA expression of NOX2 or TRPM8 (Fig. 8). NOX2 or TRPM8 expression had no effect on ER+ patient survival; however, overall survival for ER− patients was significantly different when separated by mRNA expression for both NOX2 and TRPM8. NOX2 and TRPM8 showed an inverse outcome on overall survival whereby patients with high expression of NOX2 showed greater survival (hazard ratio, 0.57; *P* = 0.0045) and patients with high expression of TRPM8 showed poor survival (hazard ratio, 1.67; *P* = 0.044). Based on this inverse relationship, the combination of NOX2 and TRPM8 mRNA expression was tested for effects on overall survival, but data were separated using inverted NOX2 expression data and unchanged TRPM8 data. The data show that the combination of low NOX2 mRNA and high TRPM8 mRNA was present in ER− patients with poor clinical outcome (hazard ratio, 2.4; *P* = 0.00072) vs. patients with NOX2 high/TRPM8 low, but also show a larger survival difference compared with the assessment of each individual gene. We further tested this same approach on other measures of clinical outcome (*SI Appendix*, Fig. S7) and found that the combination of low NOX2 mRNA and high TRPM8 mRNA similarly predicted poor outcome in ER− patients in multiple survival categories (relapse-free survival, distant metastasis-free survival, and postprogression survival).

In conclusion, we find that alterations in two major components of a mechanically-sensitive pathway identified in nontumorigenic MCF10A cells, NOX2 and TRPM8, are correlated with overall survival of ER− breast cancer patients. While changes in expression of each individual gene were associated with poor survival, the combination yielded an even larger overall survival difference (hazard ratio, 2.4). Not only can this mechanism of mechanoresponsiveness be disrupted by oncogenic activation of KRas, which occurs at the level of ROS sensing, but clinical data further suggest that dysregulation of this pathway at the level of ROS generation (NOX2) and calcium influx (TRPM8) is present in breast cancer patients and predictive of clinical outcome.

## Discussion

We report that nontumorigenic breast epithelial cells are mechanically sensitive, responding with two distinct calcium responses, an initial intracellular calcium release and a subsequent response of extracellular calcium influx that persists for up to 30 min. The persistent calcium signal results from the microtubule-dependent activation of NOX2, a ROS-generating integral membrane enzyme. The NOX2-ROS in turn acts on TRPM8 to sustain mechanically-activated calcium signaling. However, oncogenic KRas activation modifies this mechanically-induced signal. The constitutively active KRas mutation reduces the initial mechanically-activated peak calcium and severely inhibits the long-term persistent calcium signal. The data show that cancer-promoting pathways such as KRas activation can alter mechanoresponsiveness at a very early stage in the signaling pathway (in less than 30 s). Given the emerging importance of altered mechanical properties in the tumor microenvironment across longer timescales (>24 h), our work helps define the mechanobiology of much earlier signaling events. Furthermore, such alterations in rapid mechanotransduction by oncogenes could provide selective advantages to tumor cells during cancer progression. The significance of this work is further highlighted by clinical data showing that NOX2 and TRPM8 affect patient survival.

This study demonstrates microtubule-dependent mechanotransduction through NOX2-ROS and calcium signaling in breast epithelial cells, and its dysregulation with oncogene activation. While further work will seek to define the downstream targets of these signals in cancer, the discovery of this mechanotransduction pathway first in heart (32), then in skeletal muscle (34) and bone osteocytes (36), elevates the pathological significance and offers additional insights. Microtubule-dependent NOX2-ROS and TRP channel calcium influx is overactive in muscular dystrophy, which contributes to arrhythmias, cardiomyopathy, and contraction-induced injury, all functions that rely on tightly controlled intracellular calcium (32, 34, 35). Evidence that in vivo targeting of the disease-altered microtubules yielded therapeutic benefit in muscle, which ameliorated downstream pathological ROS and calcium influx, support these alterations as disease modifiers and therapeutic targets (34, 35). Further work in mechanically-sensitive bone osteocytes, reported that fluid shear stress acts through microtubules to elicit NOX2-ROS signals that drive TRPV4 calcium flux, which regulates CaMKII and the protein sclerostin [an inhibitor of bone formation (55, 56)].

While microtubules have long been chemotherapeutic targets in cancer, there is new evidence that NADPH oxidases may also play a role in breast cancer. NOX2 has been reported to contribute to cell growth and proliferation in breast (57) and other epithelial cancers (colorectal and prostate). Data on breast NOX2 expression are minimal, with some studies showing mRNA or protein expression in breast cancer cell lines (57–60) and mRNA in nontumorigenic (MCF10A) cells (59), while patient sample expression remains largely unexplored (57). However, our data support a role for the mechanical activation of NOX2 in breast epithelial cells, and we further report that KRas mutation alters this pathway. Due to the common dysregulation of the Ras/MAPK pathway in breast cancer, inhibition of NOX2-dependent mechanotransduction by oncogenic activation of KRas may play an important role in breast malignancies; however, the specific role for mechanically-activated NOX2 in breast and other epithelial cancers has yet to be defined.

There is mounting evidence implicating dysregulated calcium signaling pathways as disease modifiers in breast cancer. Calcium signaling is affected at multiple levels via calcium, calcium-permeable channels, and calcium-binding proteins and is thought to play an important role in tumor progression (reviewed in refs. 61–63). TRPM8 is part of the TRP family of calcium-permeable ion channels. TRP channels have a wide variety of activation such as heat/capsaicin, cold/menthol, stretch-activation, pH, and voltage (reviewed in refs. 64 and 65). Although TRPM8 channels are primarily thought of as cold/menthol sensing calcium-permeable channels in sensory neurons (66–68), they are also activated by voltage and phosphatidylinositol 4,5-bisphosphate (PIP2), with reductions in PIP2 desensitizing the channel (68–70), and are expressed in other tissues including the breast (49, 50). Increased expression of TRPM8 channels has been reported for breast cancer (49, 50), as well as other epithelial cancers (prostate, pancreatic, lung, gastric, melanoma; reviewed in ref. 71), and is thought to play a role in proliferation and apoptosis.

For mechanical activation of TRP channels, a large amount of data currently suggest that TRPV4 is integral to mechanosensory processes (reviewed in refs. 72 and 73), and so far TRPV4 is the only channel that has been identified in NOX2-dependent mechanical processes (36). However, it is unknown whether TRMP8 or other TRPM channels are directly or indirectly mechanoresponsive (reviewed in refs. 72 and 73). Indeed, one study suggests that TRPM channels are insensitive to direct membrane stretch (74). While it is possible that mechanical stimuli such as mSLED directly trigger the opening of TRPM8 followed by NOX2-derived ROS acting to delay channel deactivation/inactivation/desensitization and thus sustaining persistent calcium, our data at the very least support a role for ROS directly initiating intracellular calcium signaling via TRPM8 in addition to at least one other recent study supporting the notion that TRPM8 can be activated by ROS in human kidney cells (75).

Our combined TRPM8 inhibition and zero calcium data show a mechanism by which extracellular calcium influx across the plasma membrane is mediated by TRPM8 receptors in response to transient mechanical cues. While the TRPM8 inhibitor used here (RQ-00203078) is more than 350-fold more selective for TRPM8 compared to other TRP channels, it remains possible that other TRP channels could be contributing. It should also be noted that there was an unexplained effect of mSLED inducing long-distance calcium signaling in neighboring cells under zero calcium conditions, without causing a loss of membrane integrity (*SI Appendix*, Fig. S8*A*). One possible mechanism could be the initiation of active ATP signaling (e.g., release from Pannexins) under zero calcium conditions, since extracellular Apyrase blocks this effect (*SI Appendix*, Fig. S8 *B* and *C*).

The microtubule post-translational modification detyrosination has been identified as a critical component of physiologic X-ROS. Detyrosinated α-tubulin, also known as glu-tubulin or detyrosination, is the process by which the enzymatic removal of a tyrosine on α-tubulin reveals a glutamate and increases the binding of microtubule associated proteins, therefore extending the lifetime of tubulin polymers (76). This effectively stabilizes microtubules, increases cytoskeletal stiffness, and modifies the setpoint at which mechanical activation can occur (36). For cancer, detyrosination is increased in patient tumor samples, cancer cell lines, and in epithelial-to-mesenchymal transition contexts like the invasive fronts of tumors (77) and wound edges (78). Changing the abundance of detyrosination can either increase or decrease X-ROS signaling depending on the tissue type. For example in dystrophy, detyrosination is overly abundant and X-ROS signaling is amplified (34, 35), while increasing detyrosination in osteocytes decreases X-ROS (36). The role of detyrosination in tuning mechanotransduction pathways and the clear associations of detyrosination with malignant phenotypes may offer fertile ground for bridging research in these fields, especially given the recent identification of the tubulin carboxypeptidase (79, 80). As an important first step, we have now established that deregulation of KRas (which promotes survival, growth, and metastatic capacity) reduces X-ROS signaling in otherwise normal mammary epithelial cells. However, whether changes in detyrosination is causative in these observations or what advantages disrupting mechanical sensation confers to these cells has yet to be explored.

We previously reported that breast epithelial cells are responsive to mechanical scratch through the initiation of cytoplasmic calcium signaling, a mechanism both spatially and temporally regulated (33), whereby signaling away from the scratch area to neighboring cells was an intercellular communication mediated by extracellular ATP and activation of purinergic P2Y2 GPCRs, but cells at the immediate scratch edge showed persistent increases in cytoplasmic calcium from extracellular sources for up to 50 min. Our data from applying scratch on dishes of increasing stiffness isolate these mechanisms, ultimately showing that signaling to neighboring cells is also mediated by cell damage (Fig. 1 and *SI Appendix*, Figs. S2 and S3). However, a much more controlled approach to mechanically activating cells would be independent of cell damage-induced signaling. Indeed, applying “scratch” on dishes of 0.2-kPa elastic modulus in the current study results in the mechanical initiation of calcium signaling in the absence of cell damage (*SI Appendix*, Fig. S4). This more focused approach to mechanically stimulating cells, now termed mSLED, results in robust increases in cytoplasmic calcium as well as a long-term persistent signal in breast epithelial cells. In situ force mapping shows that normal breast epithelial cells resident in mammary ducts have an average elastic modulus of ∼0.2 to 0.4 kPa (9); therefore, the elastic environment of these dishes better recapitulates physiologic mammary gland mechanics. Moreover, mSLED allows us to answer questions in cancer mechanobiology on faster timescales, and as we report here, KRas oncogene activation disrupts the ability of breast epithelial cells to respond to acute extracellular mechanical cues with rapid calcium signaling.

Normal cell function relies on mechanical sensation by mechanotransducers and the ability to respond to changes in environmental mechanical signals (81–88). Typical mechanotransducers include ion channels [Piezo or transient receptor potential (TRP) channels], integrin/talin complexes, or e-cadherin/α-catenin complexes, which respond to extracellular physical cues and initiate intracellular biochemical signal cascades such as calcium signaling [Piezo (31) and (89) and TRP (reviewed in refs. 90 and 91)], focal adhesion maturation (reviewed in ref. 92), or adherens junction stabilization (reviewed in ref. 92), respectively. Moreover, complete knockouts of mechanotransducers such as piezo proteins lead to embryonic lethality in mice (93, 94), and loss- or gain-of-function mutations in humans can lead to a variety of genetic diseases (reviewed in ref. 95). Mounting evidence shows the critical functions of normal mechanosensory and mechanotransduction mechanisms, and the current study reveals that specific oncogenic mutations can disrupt mechanoresponsiveness.

In conclusion, our data support a unique mechanism by which mechanical stimulation of breast epithelial cells results in the rapid activation of NOX2 yielding ROS that stimulates the intake of extracellular calcium through TRPM8 channels (Fig. 7*D*). The role for detyrosinated microtubules, NOX2, ROS, and a TRP channel are consistent with X-ROS mechanisms described in muscle and bone. The disruption of normal mechanoresponsiveness is linked to diseases of muscle and bone, and we now link the disruption of mechanoresponsiveness to KRas oncogene activation in the breast. Moreover, we report that expression changes in NOX2 and TRPM8 are correlated with poor clinical prognosis, which suggests disruption of this mechanically-activated pathway in breast cancer patients in vivo. This clarification of the molecular mechanisms underlying rapid mechanical signaling in mammary epithelial cells will now enable studies of how other oncogenic mutations and chronic alterations to the mechanical microenvironment intersect with these pathways, which could yield new therapeutic targets for cancer.

## Supplementary Material

Supplementary File

Supplementary File

Supplementary File

## Data Availability

There are no restrictions on the availability of materials or information. Data are provided in this manuscript as well as in Dataset S1. All MATLAB analysis code used in this manuscript is publicly available in a GitHub repository (DOI: 10.5281/zenodo.3986143).
