# Supplementary material for: Mechanoactivation of NOX2-generated ROS elicits persistent TRPM8 Ca2+ signals that are inhibited by oncogenic KRas
Source: Proc Natl Acad Sci U S A. 2020 Oct 5;117(42):26008–19. doi: 10.1073/pnas.2009495117 (PMC7584994; doi:10.1073/pnas.2009495117)
Supplement: Supplementary File [file pnas.2009495117.sapp.pdf]

PNAS
www.pnas.org

Supplementary Information for

Mechanoactivation of NOX2-generated ROS elicits persistent TRPM8 Ca^{2+} signals that are inhibited by oncogenic KRas

Stephen JP Pratt[1,2,3], Rachel M Lee[2,3], Katarina T Chang[2,3], Erick O Hernández-Ochoa[4], David A Annis[2,3], Eleanor C Ory[2,3], Keyata N Thompson[2,3], Patrick C Bailey[1,2,3], Trevor J Mathias[2,3], Julia A Ju[2,3], Michele I Vitolo[2,3], Martin F Schneider[4], Joseph P Stains[5], Christopher W Ward[5,6], Stuart S Martin[2,3]

Program in Biochemistry and Molecular Biology[1], Department of Physiology[2], Marlene and Stewart Greenebaum NCI Comprehensive Cancer Center[3], Department of Biochemistry and Molecular Biology[4], Department of Orthopaedics[5], University of Maryland School of Medicine, Baltimore, MD
School of Nursing[6], University of Maryland, Baltimore, MD

Stephen JP Pratt and Stuart S Martin
Email: sjppratt@umaryland.edu, SSMartin@som.umaryland.edu

This PDF file includes:

Supplementary text
Figures S1 to S8
Legend for Movie S1

Other supplementary materials for this manuscript include the following:

Movie S1
Dataset S1

www.pnas.org/cgi/doi/10.1073/pnas.2009495117

Supplementary Information Text

Expanded manuscript methods

Cell culture

Human breast MCF10A epithelial cells were obtained from the American Type Culture Collection. Cells were regularly monitored for mycoplasma contamination and have tested negative. Cells were maintained at 37°C, 5% CO_2 in Dulbecco's Modified Eagle Medium / F12 + GlutaMAX (Gibco 10565-018) supplemented with 5% horse serum (Invitrogen 26050-088), 1% penicillin-streptomycin (Gemini Bioproducts 400-109), 20ng/ml recombinant human epidermal growth factor (Gibco PHG0311), 0.5mg/ml hydrocortisone (Sigma Aldrich H-0135), 100ng/ml cholera toxin (Sigma Aldrich C-8052), 10µg/ml insulin (Sigma Aldrich I-9278). To maintain stocks, cells were passaged using a brief wash in phosphate-buffered saline (PBS) (Quality biological 114-058-101) followed by incubation with 0.25% Trypsin and 2.21mM ethylenediaminetetraacetic acid (EDTA) (Corning 25-053-CI) at 37°C, 5% CO_2. For most experiments, cells were plated to confluency overnight in soft substrate 6-well plates containing a 0.5mm biocompatible silicone layer of elastic modulus 0.2kPa (Advanced Biomatrix CytoSoft 6-Well Plate, Elastic Modulus 0.2kPa, 5165). For a subset of experiments, cells were plated on plates containing substrates of varying rigidities (elastic moduli 0.2, 0.5, 2, 8, 16, 32, 64kPa, Advanced Biomatrix CytoSoft Discovery Kit 6-Well Plates, 5190). These commercial plates require coating with matrix proteins per manufacturer's instructions, plates were coated with 2.63µg/cm^2 Mouse Collagen IV (Corning 354233)

PTEN-/- and KRas mutations

The creation of cells and tumorigenic capacity is described in [1] and metastatic capacity in [2]. Human breast MCF10A cells were used as a non-tumorigenic breast epithelial control and two mutations were introduced (PTEN-/- and activated KRas overexpression) to mimic the deregulation of two common pathways frequently altered in breast cancer; the phosphatidylinositol 3-kinase (PI3K) pathway and the Ras/MAPK pathway. The creation of the PTEN-/- cells has been described previously [3]. Briefly, heterozygote clones were created as previously described [4] and confirmed individual single cell PTEN+/- clones were infected with an adeno-associated virus that was generated using an AAV Helper-free system to target specific intronic sequences flanking exon 2 within the PTEN gene. Individual G418-resistant clones were tested via PCR for the presence of the correct incorporation of the Neomycin resistance gene in place of the exon 2 of PTEN. Positive clones were

treated with a Cre recombinase virus to excise the IRES-neoR gene. The 10A-KRas lines were constructed by retroviral infection of the oncogenic KRas(G12V) gene. The pLXSN retroviral vector containing KRas(G12V) was a generous gift from Dr. Ben Ho Park (Vanderbilt University). Retroviral particles were generated using the AmphoPack-293 cells (Clontech) following manufacturer's instructions. MCF10A cells were infected with the retrovirus in combination with 8μg/ml polybrene and selected with 0.12μg/ml neomycin after 48 hours. Activated KRas overexpression was verified by Western blot using primary antibodies against KRas (clone 234, MilliporeSigma), phosphorylated extracellular signal-regulated kinase 1/2 (pERK1/2) (Cell Signaling), and ERK1/2 (Cell Signaling).

Reagents

Prior to imaging, cells were loaded using 4μM Fluo-4 AM (a cytosolic and calcium sensitive dye, Life Technologies F14201) in Hanks Balanced Salt Solution containing calcium (HBSS+Ca^{2+}, Gibco 14025-092) for 30 minutes and washed in 1mL HBSS+Ca^{2+} for 30 minutes. This final wash was used during imaging unless otherwise stated. To measure compromised cell plasma membranes, 1.5μM propidium iodide (PI, Sigma Aldrich P4864) was added to the final wash step. For depletion of internal cellular calcium stores, cells were treated with 2μM thapsigargin (Sigma Aldrich T9033) for 10 minutes prior to dye loading, washing, and imaging. To deplete external calcium stores, dishes were washed twice briefly in 1mL Hanks Balanced Salt Solution without calcium (HBSS-Ca^{2+}, Gibco 14025-092) supplemented with 100μM ethylene glycol-bis(β-aminoethyl ether)-N,N,N',N'-tetraacetic acid (EGTA) (Sigma Aldrich E3889) and then washed in a final 1mL HBSS-Ca^{2+} for 5 minutes prior to imaging. Apyrase (Sigma Aldrich A6535) was reconstituted to 10units/mL in HBSS+Ca^{2+} and used to cleave extracellular ATP to ADP/AMP. After dye loading and washing, dishes were incubated in 1mL Apyrase (10units) for 10 minutes prior to imaging, without washout. For introduction of reactive oxygen species (ROS) to cells, hydrogen peroxide (H_2O_2, Sigma Aldrich 21673) was diluted to a 20mM stock in HBSS+Ca^{2+} and added for a final 10mM concentration to cells during imaging of Fluo-4 fluorescence. Addition of HBSS+Ca^{2+} alone ("Media Alone") or the absence of any intervention ("No Intervention") were used as controls. To inhibit ROS, cell imaging media was replaced with 40mM N-acetylcysteine (NAC, USP Reference Standard 1009005) in Ringer's solution for 5 minutes prior to imaging. An inhibitory peptide GP91ds-TAT (Anaspec, AS-63818) that specifically blocks NOX2 activity [5], was added to the cells at 1.5μM in HBSS+Ca^{2+} for 5 minutes prior to imaging. Colchicine

(Sigma Aldrich C9754) was used to specifically depolymerize microtubules, and cell imaging media was replaced with 200μM in HBSS+Ca^{2+} for 10 minutes prior to imaging. Parthenolide (Sigma Aldrich P0667) decreases detyrosination, a microtubule stabilizing post-translational modification, and cell imaging media was replaced with 200μM in HBSS+Ca^{2+} for 10 minutes prior to imaging. In order to target transient receptor potential (TRP) channels, two non-specific TRP inhibitors were used 2-Aminoethoxydiphenylborane (2-APB) (200μM for 10 minutes, Tocris 1224) and Ruthenium Red (RR) (30μM for 30 minutes, Tocris 1439). A potent and selective transient receptor potential cation channel subfamily M member 8 (TRPM8) antagonist RQ-00203078 was used to target TRPM8 (25μM for 20 minutes, Tocris 5388). Because some stock solutions of inhibitors were reconstituted in dimethyl sulfoxide (DMSO) (Sigma Aldrich 276855), a 5% DMSO in HBSS+Ca^{2+} control was used to test for effects of DMSO. Final working concentrations of DMSO for inhibitors used in data collection were much less than 5% DMSO.

Mechanical stimulation on low elastic modulus dishes (mSLED) and dishes of increasing stiffness

mSLED is possible when cells are plated on low elastic modulus substrates (0.2kPa, Advanced Biomatrix 5165) however a subset of experiments used substrates of increasing stiffness (elastic moduli 0.2, 0.5, 2, 8, 16, 32, 64kPa, Advanced Biomatrix 5190) using identical methods otherwise. Glass capillaries (Warner Instruments Borosillicate Glass Model No: G150-4, Order No: 64-0772, Outer Diameter: 1.5mm, Inner Diameter: 0.86mm) were pulled and fire polished such that the end of the pipette tip was melted and sealed to a ~160μm rounded, bulbous end. Pipettes were attached to a motorized micromanipulator (Sutter Instrument MP-225) used to maneuver the pipette in *x*, *y*, and *z* directions. Experiments were conducted on an Olympus IX81 microscope with a Fluoview FV1000 confocal laser scanning system at room temperature. 512 x 512 pixel images with 2.485μm/pixel spatial scale were collected at 2.0μs sampling speed using a 10x objective and 0.4 numerical aperture. As visualized in light microscopy, the glass pipette was used to compress the cell monolayer outside of the confocal scan area. Confocal time series imaging was immediately initiated (200 frames at 2 second frame rate) and the pipette was maneuvered in the *x* direction until the pipette stimulus was applied across the entirety of the cell monolayer in view (similar to and adapted from a scratch assay, but does not create a scratch/gap area or induce cell damage). The pipette remained in its final place until completion of imaging. For treatment with inhibitors, the final 1mL HBSS+Ca^{2+} wash (after Fluo-4 dye loading) was replaced with 1mL HBSS+Ca^{2+}

containing the respective molar concentration of inhibitor for 5-10 minutes prior to initiation of confocal time series imaging and pipette movement. For experiments measuring 30-minute long term calcium persistence, 450 frames were collected at 4 second frame rate.

mSLED optimization and force calculations

mSLED stimulation involves both compression of the cell monolayer and simultaneous lateral movement of the pipette across the monolayer. In order to justify the degree of compression to achieve maximal calcium response, Z-stack images of mSLED stimulated cells were used to map the indentation of cell monolayer by pipette. The indentation depth of the cell monolayer was quantified using the difference between the average monolayer baseline (top) and bottom hundredth percentile of the indentation (bottom). Two depths of pipette compression (Normal vs Shallow) were tested and compared with the respective calcium responses. In addition, forces applied to cells by pipette were calculated using the indentation depth (*d*), known elastic modulus of substrate (*E* = 0.2kPa), and pipette radius (*R*) = 80 μm, using the Hertzian contact mechanics equation for a rigid sphere indenter [6] $\mathrm{F} = \frac{4}{3}\frac{\mathrm{E}}{1-\upsilon^2}\mathrm{R}^{1/2}\mathrm{d}^{3/2}$. The exact Poisson's ratio of the highly elastic 0.2kPa CytoSoft plates is unknown so here we assume $v = 0.5$ to calculate approximate forces.

ROI-based Analysis: Edge and neighbor time traces, Peak ΔF/F, ΔF/F at 6 minutes

Relative change in Fluo-4 fluorescence was calculated as ΔF/F using regions of interest (ROI). Custom scripts were written in MATLAB to automatically identify and analyze each ROI. The location of the glass pipette tip was extracted by creating binary images from the differential interference contrast (DIC) images acquired. The median location of this glass tip over time was used as the centerline of the scratch (i.e. pipette stimulus on high elastic modulus dishes) or mSLED stimulus (i.e. pipette stimulus on low elastic modulus dishes) areas for creating each ROI. In each case, a region centered on the scratch or mSLED stimulus area location with width 512 pixels (the image width) and height 100 pixels was used as the ROI for analysis of the signaling within the scratch edge or mSLED areas. All pixels in the image not contained within the edge ROI were used for calculations describing the neighboring regions away from the scratch edge (relevant for scratch only). For each ROI, the total intensity of pixels within the ROI was determined in each frame of the time lapse (200 frames for 6.7-minute videos, 450 frames for 30-minute videos). This value was converted to ΔF/F by subtracting the value of the first frame from

each frame and dividing the resulting subtracted value by the value of the first frame [$\Delta F/F = (F-F_0)/F_0$]. Peak $\Delta F/F$ was determined by finding the maximum of this curve over time. The value of the $\Delta F/F$ curve at 6 minutes (frame 180) was used as a measure of fluorescence persistence. For mSLED experiments, edge *vs.* neighbor ROI separation and analysis was no longer necessary (since neighboring signaling was absent), therefore only data from "edge" ROIs (i.e. 512x100 pixel ROI was centered around the mSLED stimulus area using the glass pipette tip) were reported. For H_2O_2 experiments, there was no ROI chosen and total intensity of pixels for the whole image was used to calculate $\Delta F/F$ as described above, for each frame in the time lapse.

ROI-independent Analysis: Distance from scratch and Kymographs

A ROI-independent analytical approach was used to investigate the distance the fluorescence signal traveled away from the scratch edge (relevant for scratch only) in which the value of $\Delta F/F$ was calculated for each distance from the scratch in the image (equivalent to the calculations described above if ROIs 1 pixel in height covered the entire image). The peak value of $\Delta F/F$ was calculated for each of these curves and plotted as function of distance from the scratch. We present the values of this curve at a distance of 60 pixels (150μm) away from the scratch centerline in bar graph format to show that the signal propagates away from the scratch area. Signals propagating away from mSLED stimulation were absent, therefore distance analysis was not necessary. To generate kymographs, intensity profiles were computed by taking the average pixel intensity along the axis of the scratch or mSLED stimulus areas (image width) for each point along the *y*-axis (image height, or perpendicular to the scratch/mSLED area) at each time point (200 frames total). The F_0 *y*-axis intensity profile was computed using the average along the *x*-axis for the first frame. Kymographs were then computed by taking the normalized *y*-axis profile [$\Delta F/F = (F-F_0)/F_0$] at each time point and displayed graphically [i.e. lining them up left (t = 0 s) to right (t = 400 s)].

Propidium Iodide Analysis

PI images were taken immediately before scratch or mSLED stimulation and just after the end of imaging using the same imaging parameters. The intensities of the initial image were subtracted from the final image on a pixel-by-pixel basis and summed to calculate the total change in intensity, ΔI. When comparing substrates of varying rigidities, ΔI was normalized by dividing by the value of ΔI at 0.2kPa from a paired experiment.

Kaplan-Meier Plotter

The online Kaplan-Meier Plotter database (https://kmplot.com/analysis/) was used to test whether NOX2 or TRPM8 mRNA expression had effects on breast cancer patient clinical outcome, specifically OS, RFS, DMFS, and PPS. The outcomes are defined as follows, OS: Overall survival, length of time from either the date of diagnosis or the start of treatment for cancer that patients diagnosed with the disease are still alive. RFS: Relapse-free survival, the length of time after primary treatment for a cancer ends that the patient survives without any signs or symptoms of that cancer. DMFS: Distant metastasis-free survival, length of time from either the date of diagnosis or the start of treatment for cancer, that patients diagnosed with the disease are free of a distant metastasis. PPS: Post-progression survival, the time elapsed between tumor progression after primary treatment and death from any cause (calculated as OS minus PFS). The database is an online tool, based on author publications [7, 8], and was established for research groups to assess the relevance of gene expression levels on the clinical outcome of cancer patients. Gene expression data was downloaded from NCBI Gene Expression Omnibus (GEO) (http://www.ncbi.nlm.nih.gov/geo/) repository and only Affymetrix HG-U133A (GPL96) and HG-U133 Plus 2.0 (GPL570) microarray were considered [7]. To probe for NOX2 or TRPM8 in breast cancer, the breast cancer KM plotter tool was selected (https://kmplot.com/analysis/index.php?p=service&cancer=breast) [7]. The Affy id/Gene symbol was then selected for either NOX2 (203923_s_at), TRPM8 (243483_at), or both ("use multiple genes"). Patient data was split by the median, the OS, RFS, DMFS, or PPS was chosen, and the analysis was restricted to subtype (i.e. ER+ *vs.* ER- status).

Statistics

Statistical analyses were performed using MATLAB. For comparison between multiple conditions, a one-way ANOVA with a post-hoc Tukey's honest difference criterion was used and significance set at $P < 0.05$. In the case of normalized ΔI values for substrates of varied rigidity, ANOVA calculations were performed on the log of $\Delta I/\Delta I(0.2kPa)$. For comparisons between pairs of conditions, a paired t-test was used. Correlations (between ΔI and either peak $\Delta F/F$ in the neighbors ROI or substrate stiffness) were calculated using the Spearman's Rho option of the MATLAB function `corr`.

[1] Thompson KN, Whipple RA, Yoon JR, Lipsky M, Charpentier MS, Boggs AE, Chakrabarti KR, Bhandary L, Hessler LK, Martin SS et al: The combinatorial activation of the PI3K and Ras/MAPK pathways is sufficient for aggressive tumor formation, while individual pathway activation supports cell persistence. Oncotarget 2015, 6(34):35231-35246.

[2] Yankaskas CL, Thompson KN, Paul CD, Vitolo MI, Mistriotis P, Mahendra A, Bajpai VK, Shea DJ, Manto KM, Chai AC et al: A microfluidic assay for the quantification of the metastatic propensity of breast cancer specimens. Nat Biomed Eng 2019, 3(6):452-465.

[3] Vitolo MI, Weiss MB, Szmacinski M, Tahir K, Waldman T, Park BH, Martin SS, Weber DJ, Bachman KE: Deletion of PTEN promotes tumorigenic signaling, resistance to anoikis, and altered response to chemotherapeutic agents in human mammary epithelial cells. Cancer Res 2009, 69(21):8275-8283

[4] Lee C, Kim JS, Waldman T: PTEN gene targeting reveals a radiation-induced size checkpoint in human cancer cells. Cancer Res 2004, 64(19):6906-6914.

[5] Rey FE, Cifuentes ME, Kiarash A, Quinn MT, Pagano PJ: Novel competitive inhibitor of NAD(P)H oxidase assembly attenuates vascular O(2)(-) and systolic blood pressure in mice. Circ Res 2001, 89(5):408-414.

[6] McKee CT, Last JA, Russell P, Murphy CJ: Indentation versus tensile measurements of Young's modulus for soft biological tissues. Tissue Eng Part B Rev 2011, 17(3):155-164.

[7] Gyorffy B, Lanczky A, Eklund AC, Denkert C, Budczies J, Li Q, Szallasi Z: An online survival analysis tool to rapidly assess the effect of 22,277 genes on breast cancer prognosis using microarray data of 1,809 patients. Breast Cancer Res Treat 2010, 123(3):725-731.

[8] Nagy A, Lanczky A, Menyhart O, Gyorffy B: Validation of miRNA prognostic power in hepatocellular carcinoma using expression data of independent datasets. Sci Rep 2018, 8(1):9227.

Supplemental Methods.

Fluid Flow Shear Stress (Fig. S4)

Human breast MCF10A epithelial cells were plated on collagen coated (2.63μg/cm^2 Mouse Collagen IV, Corning 354233) 0.2kPa 96 well plates (Advanced Biomatrix CytoSoft, 5255) and loaded with 4μM of Fluo-4 AM (Life Technologies F14201) to measure changes in cytosolic calcium. Cells were subjected to fluid flow using different

fluid injection rates of media (Hanks Balanced Salt Solution containing calcium, HBSS+Ca^{2+}, Gibco 14025-092) and relative changes in fluorescence were measured using a Molecular Devices FlexStation 3 multi-mode microplate reader. Initial volume of HBSS+Ca^{2+} in each well was 25μl and 50μl of HBSS+Ca^{2+} injected for a final volume of 75μl. Rates of fluid flow were: 16, 47, 78 and 125 μl/second. With fluid flow initiated at 25 seconds, fluorescence was measured every 1.3 seconds from 0-395 seconds.

Western Blot (Fig. S6)

Cells were harvested in lysis buffer supplemented with protease inhibitor cocktail, and protein concentrations determined. Lysates were equally loaded and protein separated by gel electrophoresis. Immunoblots were probed using anti-TRPM8 (1:1000, Abcam 3243, 125kDa) and anti-GAPDH.

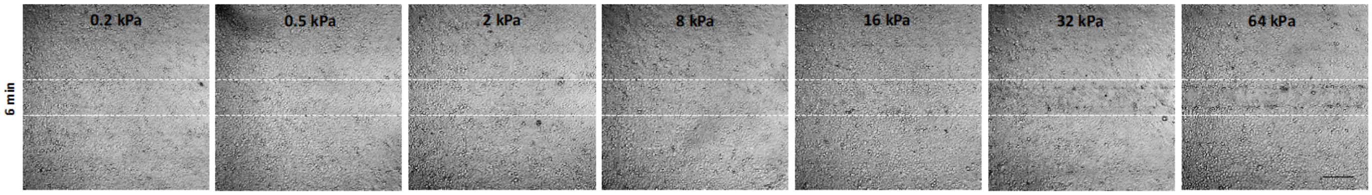

Fig. S1. DIC images of scratch on dishes of increasing stiffness.

Differential interference contrast (DIC) images of MCF10A breast epithelial cells plated on dishes of increasing stiffness (elastic modulus 0.2-64kPa) were taken 6 minutes after being mechanically scratched using a blunt fire-polished glass microprobe. Within the scratch area (dotted lines), the appearance of a scratch and cellular damage is observed on the stiffest dishes (32 and 64kPa), while the scratch area on the softest dishes (0.2 and 0.5kPa) is no different from the rest of the monolayer. Scale bar equals 200μm.

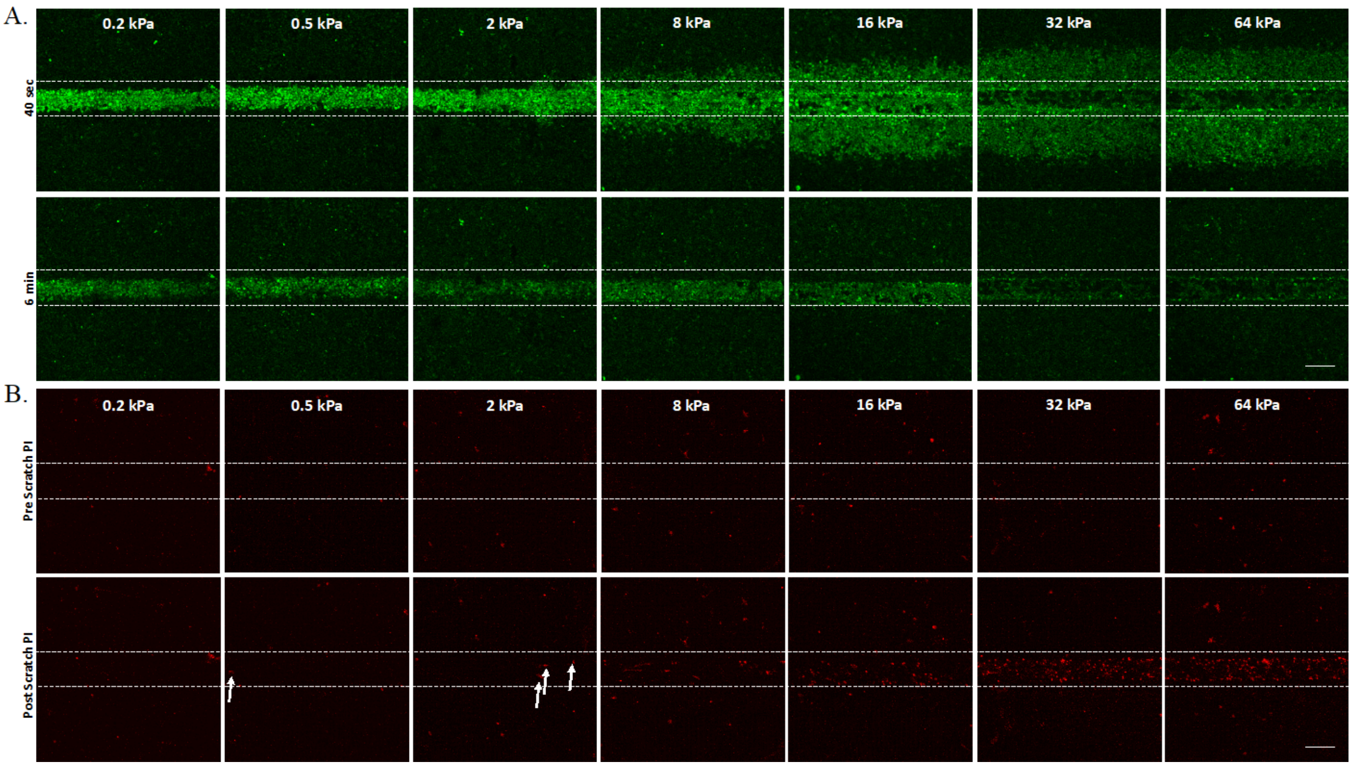

Fig. S2. Scratch-induced cell signaling to neighbors is dependent on environmental stiffness.

A: Human breast epithelial MCF10A cells were loaded with the calcium sensitive dye Fluo-4, plated on dishes of increasing stiffness (elastic modulus 0.2-64kPa) and were mechanically scratched using a blunt fire-polished glass microprobe. At any given stiffness, scratch-induced rapid rises in intracellular calcium (40s) were observed inside the scratch area (dotted lines), which persisted at later timepoints (6min). In contrast, increasing substrate rigidities resulted in a wave-like pattern of calcium signaling in neighboring cells propagating away from the scratch area. Qualitative observation shows that the distance of the propagation of the calcium in neighboring cells increases as the cellular environment gets stiffer. **B:** The absence of Fluo-4 signal in the scratch area suggested that the cells were ripped away or damaged, therefore propidium iodide (PI) was added to the extracellular solution to measure cell death or damage to cellular membranes. Post-scratch images were compared to pre-scratch images and show an accumulation of PI staining specifically in the scratch area with increasingly stiff substrates. Increases in PI staining and calcium signaling in neighboring cells away from the scratch area was an effect primarily present in the stiff dishes but not in the soft dishes and occur in parallel, thus cell damage likely mediates intercellular signaling. Scale bars equal 200μm.

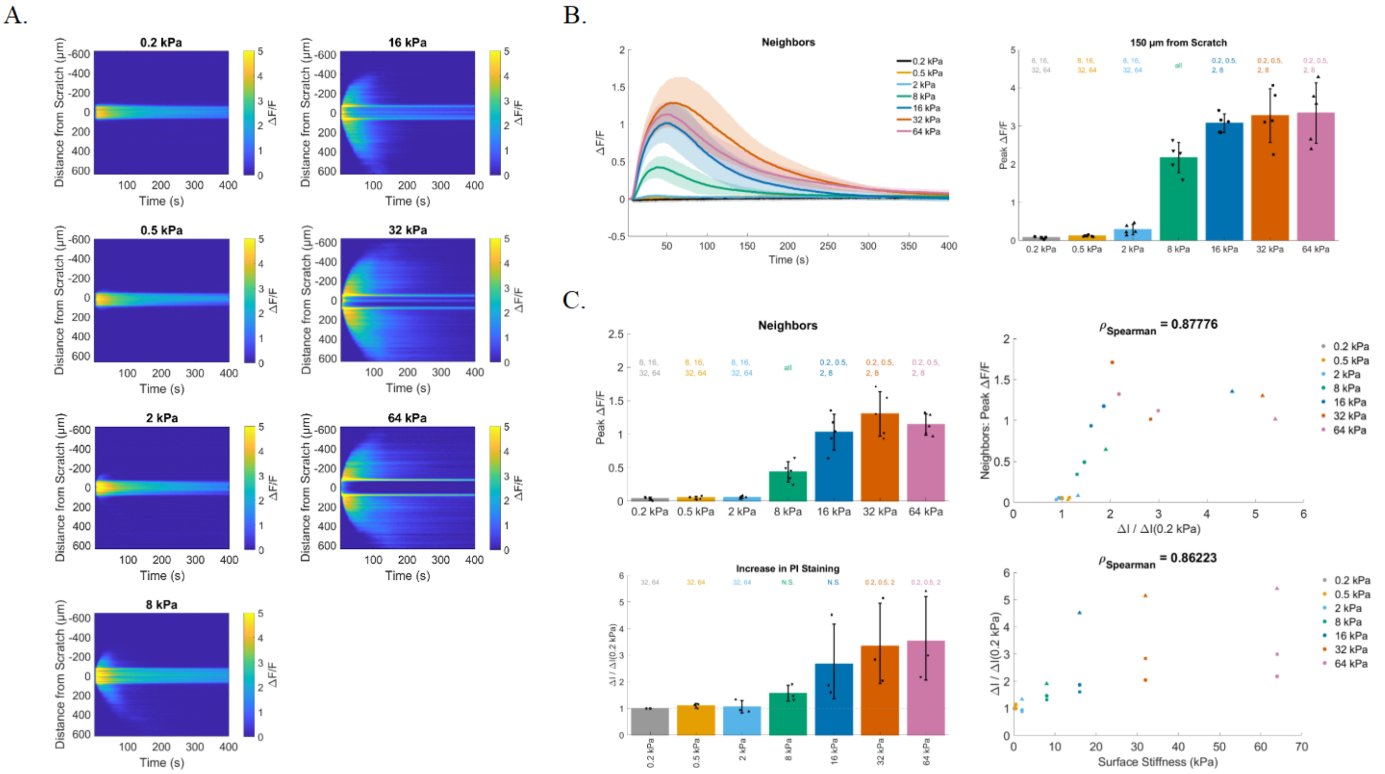

Fig. S3. Cell damage is necessary for signaling to neighbors.

A: Kymographs were generated using *y*-axis time projections at each time point over 400 seconds and show total ΔF/F signaling across the cell monolayer (0 ± 600μm) as well as within the scratch area (0 ± 125μm). Kymographs show the presence of signaling away from the stimulus area when cells were stimulated by the pipette on the stiffest substrates (8-64kPa) but not the softest substrates. **B:** In addition to ROI-independent kymographs, the relative change in Fluo-4 fluorescence was calculated as ΔF/F using automated regions of interest (ROI). ROI dimensions were set at 1.3mm (image width) by 250μm (125μm on either side of the scratch center line) and were used to quantify changes in calcium from cells within the scratch area (pixels within ROI) *vs.* that of neighboring cells (remaining pixels in image). In agreement with kymographs, quantification of total neighboring cell fluorescence (ΔF/F) confirm the increasing calcium signaling from neighboring cells as substrate stiffness increases (*left panel*). Analysis also showed that signaling in neighboring cells (Peak ΔF/F) reached longer distances (150μm from scratch area) only on the stiffest substrates (8-64kPa) (*right panel*). **C:** Peak ΔF/F in neighboring cells for each substrate stiffness (*top left panel*) was compared to total change in propidium iodide fluorescence (ΔI, post-scratch/pre-scratch) relative to 0.2kPa as a baseline (ΔI/ΔI(0.2kPa)) (*bottom left panel*).

Both Peak ΔF/F and ΔI/ΔI(0.2kPa) show a gradual increase in signal from cells subjected to scratch on the softest to stiffest dishes. The appearance of the bar graphs strongly suggested relationships between ΔI/ΔI(0.2kPa) *vs.* ΔF/F in neighboring cells *vs.* substrate stiffness (0.2-64kPa). Spearman correlations were therefore calculated for ΔF/F in neighboring cells *vs.* ΔI/ΔI(0.2kPa) (*top right panel*) and ΔI/ΔI(0.2kPa) *vs.* substrate stiffness (*bottom right panel*) and were 0.88 and 0.86 respectively. The high correlation (0.86) between substrate stiffness and PI-staining, as well as a high correlation (0.88) between PI-staining and calcium signaling in neighboring cells indicates that loss of membrane integrity occurs in the mechanically scratched area on stiffer surfaces and generates a damage-dependent signal necessary for activation of calcium in neighboring cells. When cells plated on soft (0.2kPa) substrates are stimulated in a manner that resembles a 'scratch wound assay', the stimulus does not create a scratch/gap area or induce cell damage-dependent signaling to neighboring cells, therefore resulting in a damage-independent calcium response. Data are presented as mean ± standard deviation. Significance was set to $P < 0.05$ via one-way ANOVA with a post-hoc Tukey's honest difference criterion. For any given elastic modulus (i.e. each bar in the bar graph), significance is noted using numbers corresponding to the elastic modulus it is different from. N.S. indicates no significant difference. 'All' indicates the value is significantly different from all other values. For PI staining, data represent N=3 in total from 3 independent experiments for each group. For calcium signaling (ΔF/F), data represent N=5 in total from 5 independent experiments for each group.

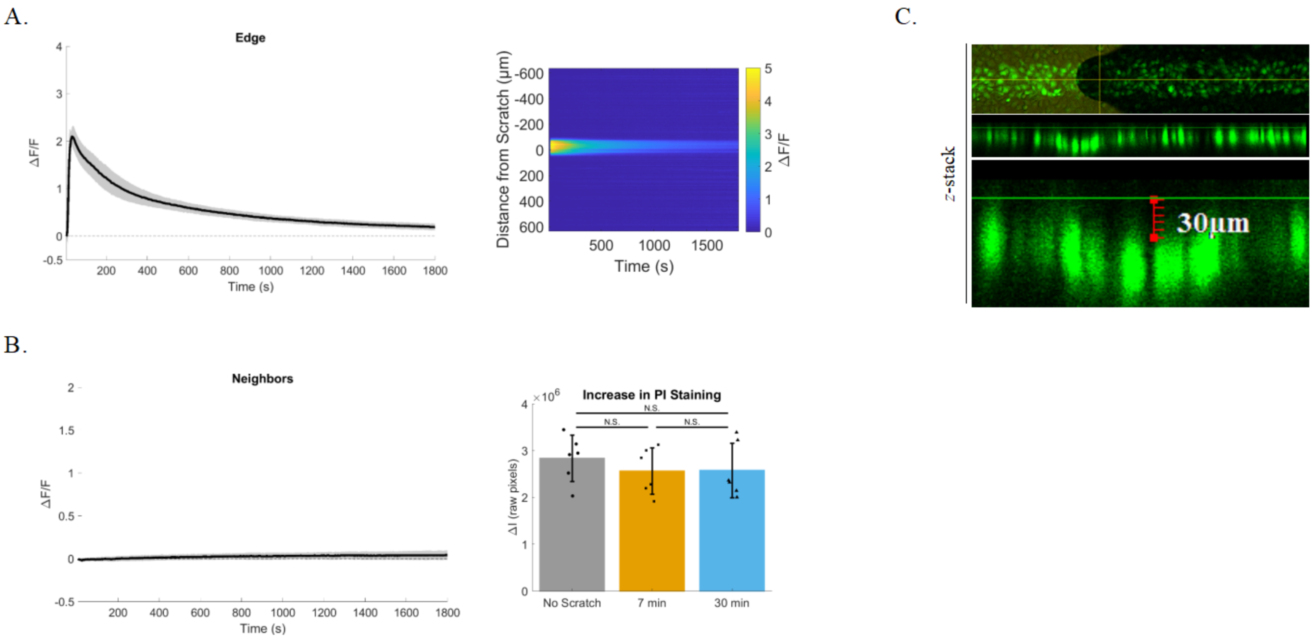

Fig. S4. mSLED: Mechanical Stimulation on Low Elastic Modulus Dishes.

A: Mechanical scratch was applied to cells plated on dishes of low elastic modulus (0.2kPa) using a 160μm wide fire-polished glass microprobe. Edge trace shows calcium signaling from cells within the stimulus area (ΔF/F) plotted over a 30-minute (1800 seconds) time course. Data displayed in the time trace and kymograph and show that the majority of signaling in response to mechanical scratch on low elastic dishes occurs within this time period. **B:** Neighbors trace shows a lack of damage-dependent signaling to neighboring cells outside of the stimulus area, suggesting cells were not damaged with mechanical scratch. Propidium iodide staining quantification (*right panel,* bar graph) confirms the absence of short-term cellular damage (7min) as well as absence of damage for the remainder of the 30-minute time course (30min). **C:** Confocal Z-stacks were used to visualize the depth of the mechanical stimulus and revealed that the cell monolayer is compressed during scratch (*z-stack*). The mechanical scratch, or mechanical stimulation, of cells plated on dishes of low elastic modulus in the absence of cellular damage is a novel method of mechanically-stimulating cells. Thus, mechanical stimulation therefore resembles a 'scratch wound assay', but does not induce a cell damaging wound (i.e. similar to and adapted from a scratch assay, but does not create a scratch/gap area or induce cell damage). We term this Mechanical Stimulation on Low Elastic Modulus Dishes (mSLED). Data presented as mean ± standard deviation. Significance was set to $P < 0.05$ via one-way ANOVA with a post-hoc Tukey's honest difference criterion. Data represent N=6 in total from 6 independent experiments for each group.

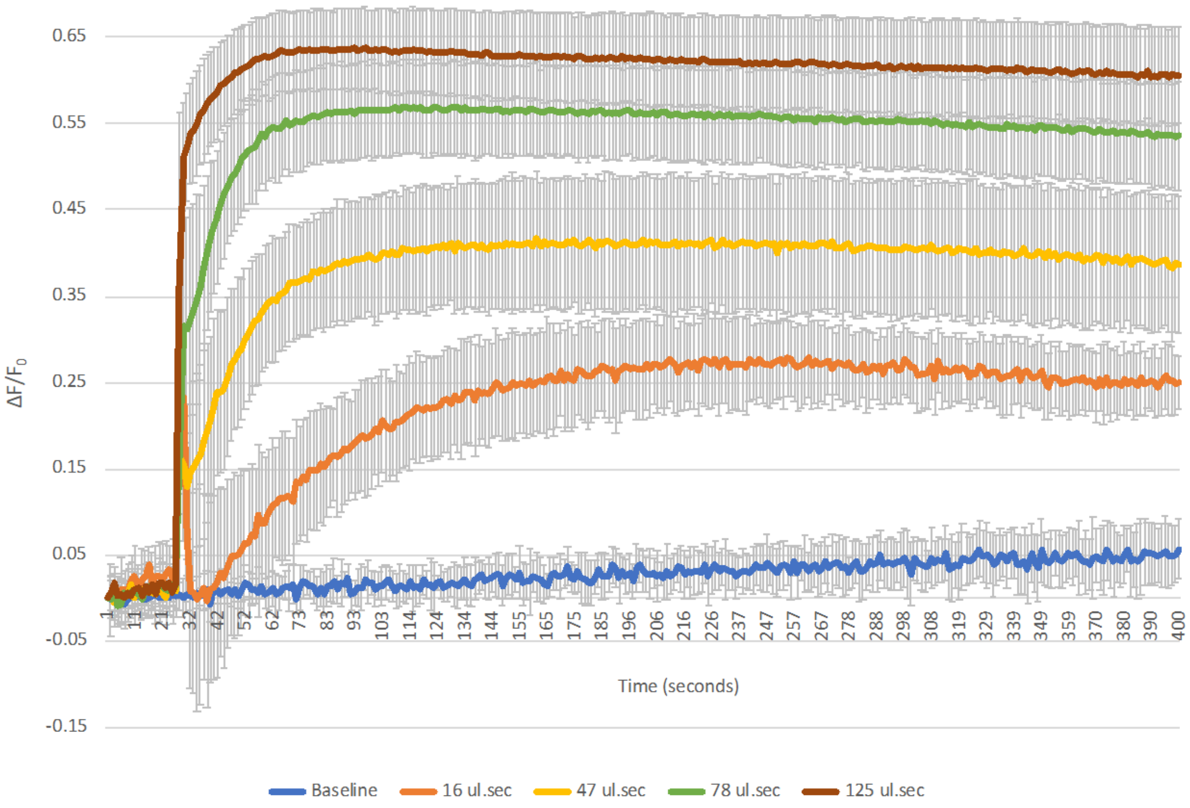

Fig. S5. Mammary epithelial cells respond to fluid flow with calcium signaling.

MCF10A breast epithelial cells were plated on 0.2kPa 96 well plates and loaded with the calcium sensitive dye Fluo-4 to measure changes in cytosolic calcium. Relative changes in fluorescence (ΔF/F) were measured using a Molecular Devices FlexStation 3 multi-mode microplate reader. With fluid flow initiated 25 seconds, fluorescence was measured every 1.3 seconds from 0-320 seconds. The rates of fluid flow were: 16, 47, 78 and 125 µl/second. Initial volume of HBSS+Ca^{2+} in each well was 25µl and 50µl of HBSS+Ca^{2+} injected for a final volume of 75µl. Compared with baseline (no injection), cells responded to different injection rates of media with the rapid initiation of calcium signaling. The data display a dose-response relationship between flow rate and peak calcium. For each curve, data points represent an average of 8 replicates.

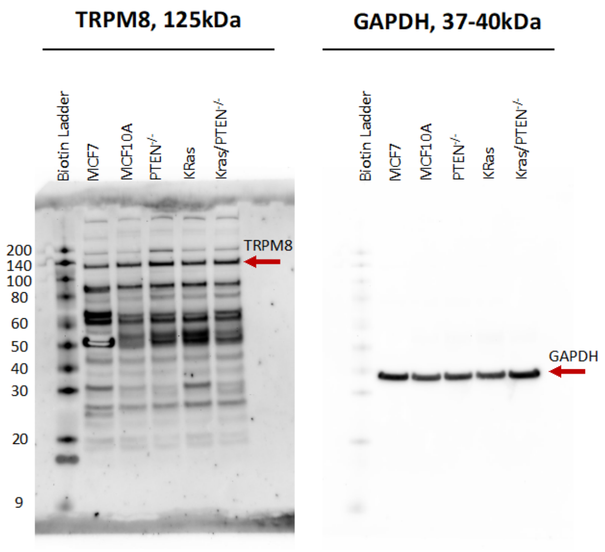

Fig. S6. TRPM8 protein expression is unchanged in MCF10A variant cell lines.

TRPM8 protein expression was probed for in MCF-7 breast cancer cells, human breast epithelial cells (MCF10A), MCF10A PTEN null mutant cells (PTEN-/-), MCF10A constitutively-active KRas(G12V) mutant cells (KRas), or MCF10A combination mutant cells (KRas/PTEN-/-). Proteins were separated by gel electrophoresis and TRPM8 expression determined using antibodies against TRPM8 (1:1000, Abcam 3243, 125kDa). Relative to the loading control (GAPDH), there are no discernible differences in TRPM8 protein expression between groups.

NOX2inv/TRPM8 mRNA Expression - Different Measures of Clinical Outcomes

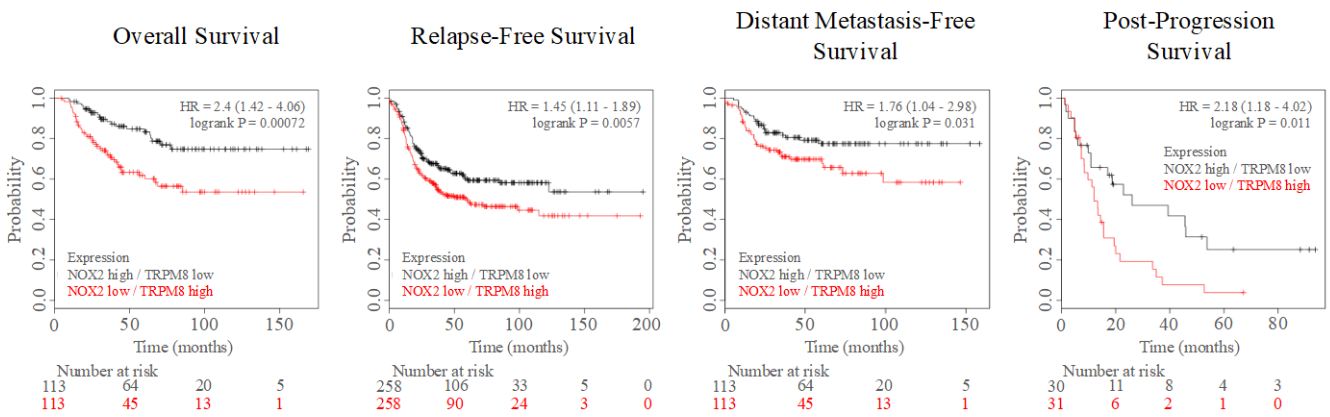

Fig. S7. NOX2 and TRPM8 mRNA expression affect estrogen receptor negative patient median OS, RFS, DMFS, and PPS.

The online Kaplan-Meier Plotter database was used to probe for NOX2 and TRPM8 mRNA expression in breast cancer estrogen receptor negative (ER-) patient clinical outcome. Overall Survival (OS), Relapse-Free Survival (RFS), Distant Metastasis-Free Survival (DMFS) and Post-Progression Survival (PPS) were compared. The combination of NOX2 and TRPM8 mRNA expression was tested for effects on OS, RFS, DMFS, and PPS, but data were separated using inverted NOX2 expression data and unchanged TRPM8 data. The data show that the combination of low NOX2 mRNA and high TRPM8 mRNA was present in ER- patients with poor clinical outcome in each of the outcome measures *vs.* patients with NOX2 high / TRPM8 low (OS Hazard Ratio = 2.4, RFS Hazard Ratio = 1.45, DMFS Hazard Ratio = 1.76, PPS Hazard Ratio = 2.18). OS: Overall survival, length of time from either the date of diagnosis or the start of treatment for cancer that patients diagnosed with the disease are still alive. RFS: Relapse-free survival, the length of time after primary treatment for a cancer ends that the patient survives without any signs or symptoms of that cancer. DMFS: Distant metastasis-free survival, length of time from either the date of diagnosis or the start of treatment for cancer, that patients diagnosed with the disease are free of a distant metastasis. PPS: Post-progression survival, the time elapsed between tumor progression after primary treatment and death from any cause (calculated as OS minus PFS). The Affy id/Gene symbol used for NOX2 and TRPM8 are (203923_s_at) and (243483_at), respectively. Patient data was split by the median. Number at risk refers to the number of patients included in each timepoint.

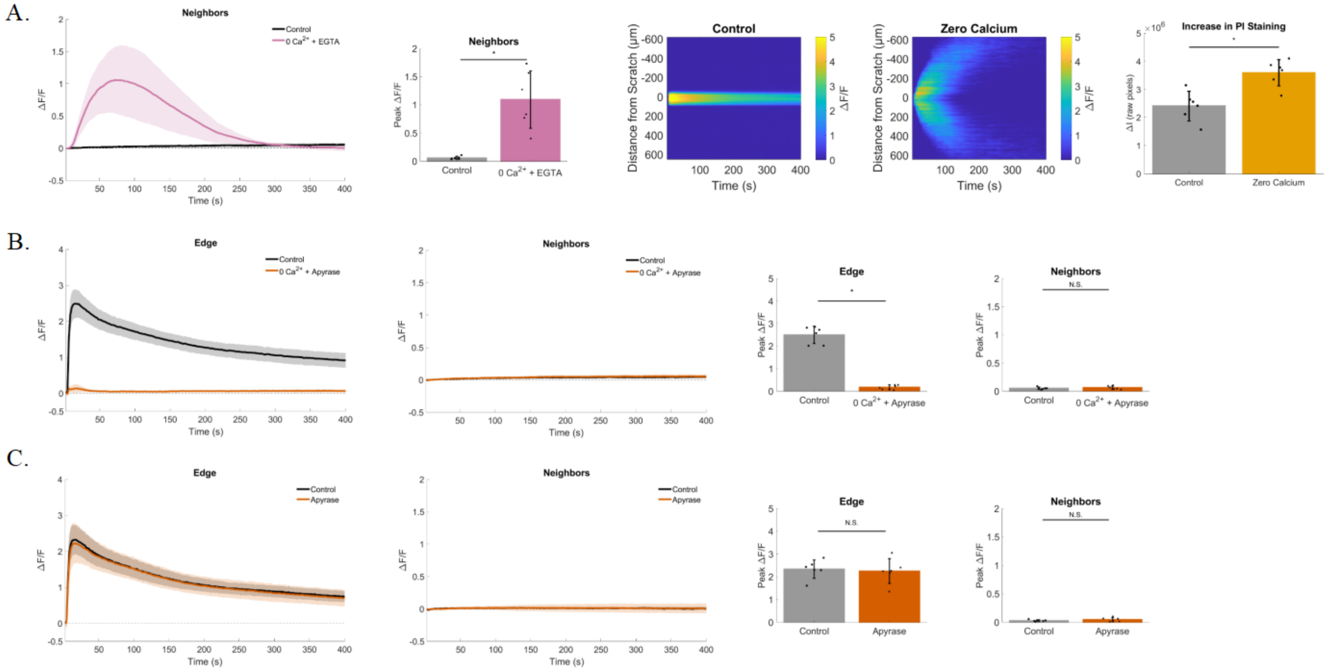

Fig. S8. Mechanical stimulation under zero extracellular calcium conditions activates signaling to neighbors but is blocked by Apyrase.

The mSLED assay was applied to MCF10A breast epithelial cells under control (with calcium) and zero extracellular calcium conditions (calcium-free imaging media supplemented with EGTA). Traces show ΔF/F plotted over time. Bar graphs show quantification of Peak ΔF/F (relative changes in Fluo-4 signal) or ΔI (change in propidium iodide staining). Edge refers to signaling within the mSLED area, while neighbors refers to signaling outside of the mSLED area. **A:** Compared with control, cells subjected to mSLED in zero calcium showed a large propagation of ΔF/F signal in cells away from the mSLED area (neighbors trace and neighbors bar graph). The distance of signal in neighboring cells was also striking (kymographs). mSLED is a non-damaging mechanical stimulation of cells, however signaling to neighbors under zero calcium conditions implied cell damage-induced intercellular signaling away from the mSLED area. Therefore, Propidium Iodide (PI) was added to the cell media to measure damage after mSLED and compared with pre-mSLED PI staining (ΔI). Compared with control, there was a small, but significant, increase in PI staining under zero calcium conditions. **B:** Zero calcium mSLED assays were applied to cells in the presence of Apyrase (enzyme that coverts ATP to ADP/AMP) to block ATP-mediated purinergic signaling and were compared with control (with calcium, without Apyrase). Apyrase completely blocked the zero calcium-induced signaling to neighbors, suggesting that ATP-dependent intercellular

communication mediates the signaling to neighbors under zero calcium conditions. **C:** Compared with control, Apyrase alone (with calcium) had no effect on mSLED-stimulated cells (edge) or cells away from the mSLED area (neighbors). Data presented as mean ± standard deviation. *indicates significance from control, $P < 0.05$ via paired t-test. Data represent N=6 in total from 6 independent experiments for each group. Control groups were collected separately for each data set.

Movie S1 (separate file). mSLED: Mechanical Stimulation on Low Elastic Modulus Dishes.

Human breast epithelial MCF10A cells were plated on low elastic (0.2kPa) substrates, loaded with the calcium sensitive dye Fluo-4 and mechanically-stimulated using a blunt fire-polished glass microprobe. Mechanically-activated calcium is independent of cellular damage. Scale bar equals 200μm.

Dataset S1 (separate file). Underlying figure data.

This spreadsheet contains the data underlying the main text and supplemental figures in a tabular format.
